# *Pseudomonas aeruginosa* Lipoxygenase LoxA Contributes to Lung Infection by Altering the Host Immune Lipid Signaling

**DOI:** 10.3389/fmicb.2019.01826

**Published:** 2019-08-14

**Authors:** Eric Morello, Teresa Pérez-Berezo, Chloé Boisseau, Thomas Baranek, Antoine Guillon, Déborah Bréa, Philippe Lanotte, Xavier Carpena, Nicolas Pietrancosta, Virginie Hervé, Reuben Ramphal, Nicolas Cenac, Mustapha Si-Tahar

**Affiliations:** ^1^INSERM, Centre d’Etude des Pathologies Respiratoires (CEPR), UMR 1100, Tours, France; ^2^Université de Tours, Tours, France; ^3^Institut de Recherche en Santé Digestive, Université de Toulouse, INSERM, INRA, Ecole Nationale Vétérinaire de Toulouse, Toulouse, France; ^4^CHRU de Tours, Service de Bactériologie-Virologie, Tours, France; ^5^Université de Tours, UMR1282 ISP, Faculté de Médecine, Equipe Bactéries et Risque Materno-Foetal, Tours, France; ^6^Institut de Biologia Molecular de Barcelona, Parc Científic de Barcelona, Barcelona, Spain; ^7^XALOC Beamline, ALBA Synchrotron, Cerdanyola del Vallès, Spain; ^8^Plateau 2MI, CNRS UMR8601, Laboratoire de Chimie et de Biochimie Pharmacologiques et Toxicologiques, Centre Universitaire des Saints-Pères, Paris, France; ^9^Université Paris Descartes, Sorbonne Paris Cité, Paris, France; ^10^Department of Medicine, University of Florida, Gainesville, FL, United States

**Keywords:** *Pseudomonas aeruginosa*, lipoxygenase, lungs, infection, lipid mediators, inflammation

## Abstract

*Pseudomonas aeruginosa* is an opportunistic bacteria and a major cause of nosocomial pneumonia. *P. aeruginosa* has many virulence factors contributing to its ability to colonize the host. LoxA is a lipoxygenase enzyme secreted by *P. aeruginosa* that oxidizes polyunsaturated fatty acids. Based on previous *in vitro* biochemical studies, several biological roles of LoxA have been hypothesized, including interference of the host lipid signaling, and modulation of bacterial invasion properties. However, the contribution of LoxA to *P. aeruginosa* lung pathogenesis *per se* remained unclear. In this study, we used complementary *in vitro* and *in vivo* approaches, clinical strains of *P. aeruginosa* as well as lipidomics technology to investigate the role of LoxA in lung infection. We found that several *P. aeruginosa* clinical isolates express LoxA. When secreted in the lungs, LoxA processes a wide range of host polyunsaturated fatty acids, which further results in the production of bioactive lipid mediators (including lipoxin A_4_). LoxA also inhibits the expression of major chemokines (e.g., MIPs and KC) and the recruitment of key leukocytes. Remarkably, LoxA promotes *P. aeruginosa* persistence in lungs tissues. Hence, our study suggests that LoxA-dependent interference of the host lipid pathways may contribute to *P. aeruginosa* lung pathogenesis.

## Introduction

*Pseudomonas aeruginosa* is a major opportunistic pathogen in humans. This Gram-negative bacterium is a major cause of morbidity in patients with dampened immune defenses. *P. aeruginosa* is also notable for its high rate of mortality in pulmonary infections (Foweraker, 2009; Fujitani et al., 2011; Sun et al., 2011). The ability of *P. aeruginosa* to proliferate in host tissues and to overcome immune defenses is largely attributable to its virulence factors secreted *via* both type 2 (T2) and type 3 (T3) secretion systems (SS) (Engel and Balachandran, 2009; Hauser, 2009; Williams et al., 2010). These secreted factors include several enzymes that are involved in host lipid metabolism (e.g., lipases, phospholipases, and epoxide hydrolase) that can act on the immune system, leading to tissue damages, and aggravated disease outcome (Bleves et al., 2010; Flitter et al., 2017).

Lipoxygenases (LOXs) are lipid-oxidizing enzymes that have long been considered to exist only in eukaryotic organisms (Porta and Rocha-Sosa, 2001; Vance et al., 2004; Hansen et al., 2013; Horn et al., 2015). In humans, six LOXs isoforms exist which can catalyze the dioxygenation of polyunsaturated fatty acids (PUFAs), including linoleic acid (LA), arachidonic acid (AA), and docosahexaenoic acid (DHA) (Ivanov et al., 2015; Kuhn et al., 2015; Mashima and Okuyama, 2015). Thus, LOXs convert PUFAs into eicosanoids (e.g., leukotrienes, lipoxins) and docosanoids (e.g., maresins, protectins, and resolvins), all major molecules with potent immunoregulatory properties (Tam, 2013; Dennis and Norris, 2015).

Interestingly, *P. aeruginosa* itself can trigger the release of PUFAs from host cell membranes, mostly through a phospholipase A2-dependent mechanism (Hurley et al., 2006; Tamang et al., 2012). Extensive *in vitro* biochemical studies characterized a specific *P. aeruginosa* 15-LOX enzyme (namely LoxA) that has the ability to oxidize both free AA and other membrane-associated PUFAs to specifically release 15-LOX products (regarding to the carbon specificity on which the oxidation occurs on AA). Involvement of LoxA in interference of host lipid signaling and cellular membrane disruption has been proposed (Vance et al., 2004; Garreta et al., 2013; Banthiya et al., 2015). Recent studies also showed that LoxA is induced during biofilm growth (Deschamps et al., 2016) and could contribute to *P. aeruginosa* pathogenesis in CF patients, by inducing ferroptosis, a cell death program involving oxidation of host cell phospholipids (Dar et al., 2018). However, the contribution of LoxA in lung infection and immune signaling *per se* awaited a thorough investigation.

In our study, we show that clinical isolates of *P. aeruginosa* do secrete a functional lipoxygenase. We also demonstrate that LoxA expression by *P. aeruginosa* in a mouse model increases the production of 15-LOX products and can modulate the host immune response during acute pneumonia. By using a recombinant form of *P. aeruginosa* LoxA, we also reveal that LoxA cooperates with immune cells to increase the biosynthesis of lipoxin A4 (LXA_4_), a key pro-resolving mediator that counter-regulates the host pro-inflammatory response and stimulates the resolution and tissue repair process (Serhan et al., 2008). Finally, we show that LoxA confers a survival advantage as it reduces *P. aeruginosa* clearance in lung tissues in our mouse model settings. Hence, our study suggests a novel mechanism by which *P. aeruginosa* may fine-tune the antimicrobial defense by interfering with the host lipid immune pathways.

## Results

### Expression of *loxA* Gene Depends on Strains and Growth-Phase of *P. aeruginos*a

**FIGURE 1:**
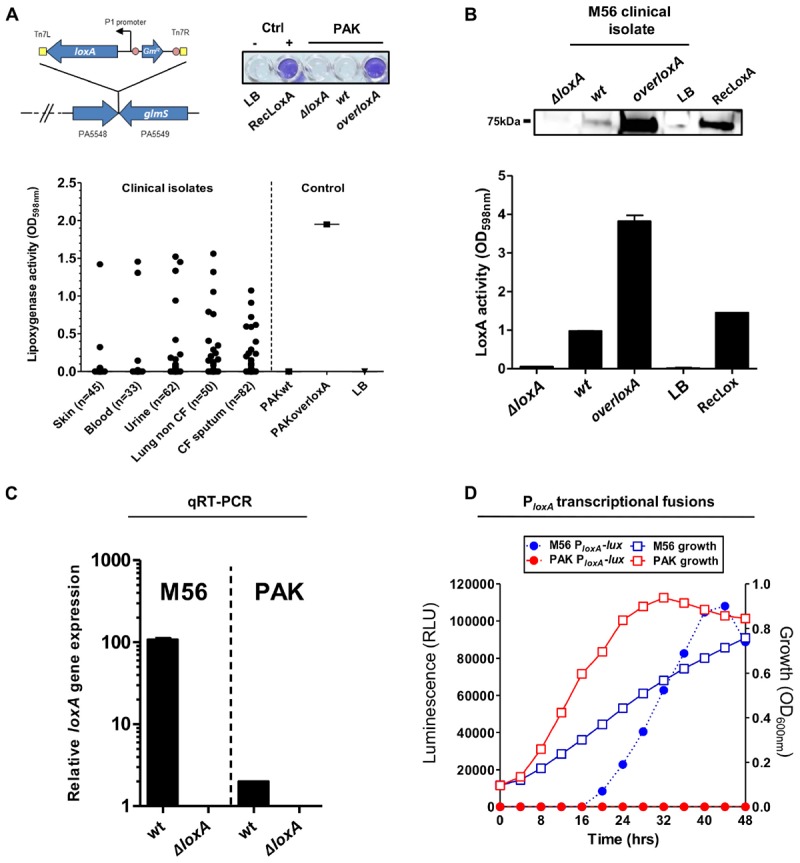
Functional lipoxygenase LoxA is secreted by *P. aeruginosa* clinical isolates through *loxA* gene expression. **(A)** Colorimetric detection of lipoxygenase activity in cultures of *P. aeruginosa* strains. Left upper panel: schematic representation of the strategy used to construct *overloxA* mutants: a constitutive expression cassette of *loxA* gene under the control of the constitutive integron promoter P1 was inserted in the chromosomal *att* site (intergenic region located between genes PA5548 and PA5549 on the chromosome) using pUC18-miniTn7T vector strategy. Upper right panel: colorimetric assay performed on overnight grown cultures (50 μl) of PAK*wt*, *ΔloxA*, and *overloxA* mutants; recombinant LoxA (RecLoxA, 100 ng) from *Pseudomonas aeruginosa* strain 42A2 was included as a positive control. Lower panel: lipoxygenase activity measured in static stationary growth-phase cultures of distinct clinical isolates of *P. aeruginosa*. Pigmented cultures could potentially biased this colorimetric assay and have been therefore excluded from these results; PAK*wt* and PAK*overloxA* were included as a positive and negative control, respectively. **(B)** Lipoxygenase activity measured in culture supernatants of M56, a LoxA-positive clinical isolate. Protein extracts from static stationary phase cultures of M56 were separated by SDS-PAGE and transferred onto nitrocellulose membranes for western immunoblotting using a rabbit anti-LoxA antibody. The specific signal corresponding to LoxA protein was correlated to lipoxygenase activity, using the same culture extracts. Samples of M56*ΔloxA* and complemented M56*overloxA* strains were used as negative and positive control, respectively. **(C)** Quantitative real-time PCR analysis of mRNA extracted from static stationary phase cultures of M56*wt* and PAK*wt*. Signal measured in *ΔloxA* mutant was considered as the background noise. **(D)** Dynamic LoxA promoter activity and growth of M56-P*_*loxA*_*-*lux* or PAK-P*_*loxA*_*-*lux* in liquid cultures by luminescence and absorbance measurements, respectively. Colonies isolated from fresh LB-agar plates were inoculated into 4 mL of LB medium (initial OD_600 nm_ 0.1) into 6-well plates. Optical densities and luminescence emission were monitored during 48 h at 28°C under static conditions, using a Tecan M200 luminometer/spectrophotometer.

The first study on LoxA by Vance and collaborators did not report any promoter activity of *loxA* gene neither in *P. aeruginosa* reference laboratory strains nor in clinical isolates (Vance et al., 2004). However, further independent studies did detect *loxA* (PA1169) gene expression in various strains especially in biofilm growth conditions (Starkey et al., 2009; Wurtzel et al., 2012; Deschamps et al., 2016). These discrepancies regarding *loxA* expression prompted us to analyze the ability of *P. aeruginosa* to secrete a functional lipoxygenase. To do so, we first adapted an assay (Anthon and Barrett, 2001) to look for lipoxygenase activity in a culture medium of the laboratory wild-type strain PAK (coined here as “PAK*wt*”), in static stationary growth-phase. The LoxA-deficient (“PAK*ΔloxA*”) strain was used as a negative control while a complemented strain that constitutively expresses *loxA* gene (“PAK*overloxA*”) was designed as a positive control. As illustrated in Figure 1A (*upper panel*), our colorimetric assay showed the typical positive blue color only in the PAK*overloxA* culture and not in the culture of PAK*wt* strain. We next screened a panel of 272 clinical isolates from various infection sites by using our colorimetric lipoxygenase assay on stationary phase culture condition, to promote biofilm formation. This included 132 *P. aeruginosa* isolates from pulmonary tract infections: 50 from non-cystic fibrosis (CF) and 82 from CF patients, 62 isolates from urinary tract infections, 45 isolates from cutaneous infections, and 33 isolates from blood. As shown in Figure 1A (*lower panel*), lipoxygenase activity was detected in 2 out of the 45 isolates from cutaneous infection (4.4%), 14 out of the 62 isolates from urinary infection (22.0%), 17 out of the 50 isolates from lungs of non-CF patients (34.0%), 15 out of the 82 isolates from lungs of CF patients (18.3%), and 3 out of the 33 isolates from blood (9.1%). LoxA protein expression was confirmed by western-blotting using a CF patient *P. aeruginosa* isolate (referred as “M56,” Figure 1B). Deletion (“M56Δ*loxA*”) and complemented (“M56*overloxA*”)-derived mutants were used as negative and positive controls, respectively. As shown in Figure 1B (*upper panel)*, compared to the M56Δ*loxA* mutant, a specific 75 kDa band was detected in samples of M56*overloxA* and to a lesser extent in “wild-type” M56. Moreover, detection of this band correlated with lipoxygenase activity assessed in the same supernatants (Figure 1B, *lower panel*). To confirm that such activity was directly related to *loxA* gene expression, we performed a qRT-PCR analysis of the M56 strain as well as of the LoxA-negative PAK*wt* strain (as a control). As shown in Figure 1C, *loxA* gene mRNA was detected only in the M56 isolate. Nevertheless, *loxA* transcripts could also be detected in the PAK strain, although to a much lower extent. Next, we monitored *loxA* gene promoter activity in both PAK and M56 strains, using a transcriptional fusion analysis and the miniCTX-*luxCDABE* vector reporter system. As shown in Figure 1D, no luciferase activity was measured in the PAK strain culture but in the M56 strain culture. This activity increased from 16 h up to 40 h post-culture and decreased afterward, indicating that *loxA* gene expression is growth phase-dependent. Of note, no lipoxygenase activity could be detected in the M56 strain when grown at 37°C, suggesting that *loxA* expression is temperature sensitive and preferentially expressed at temperature lower than 37°C (Supplementary Figure S1). Hence, our results show that *P. aeruginosa* clinical isolates can produce an active lipoxygenase and that the expression of the *loxA* gene is growth phase- and temperature sensitive.

### LoxA Secreted by *P. aeruginosa* Increases the Production of 15-LOX-Dependent Metabolites in Lung Epithelial Cells

To assess LoxA effects on the host response, we quantified LOX products in human lung epithelial NCI-H292 cells challenged by *P. aeruginosa* expressing or not the *loxA* gene. M56 strain could not be used in these specific experiments due to its high cytotoxicity (data not shown). Accordingly, we used the less cytotoxic *P. aeruginosa* PAKΔ*pscf*Δ*fliC* strain (referred hereafter as PAK’) since it was previously shown to have appropriate properties to reveal the specific role of T2SS-dependent virulence factors in both *in vitro* and *in vivo* infection models (Jyot et al., 2011). Moreover, as we previously found that PAK strain is constitutively an extremely weak expresser of *loxA* gene (Figures 1A,D), we generated a PAKΔ*pscf*Δ*fliC*-derived *overloxA* mutant (hereafter referred as PAK’*overloxA*). Importantly, isolates harboring distinct mutations (including in the promoter sequence) with a sustained expression of *loxA* have already been described (Kresse et al., 2006; Starkey et al., 2009).

**FIGURE 2:**
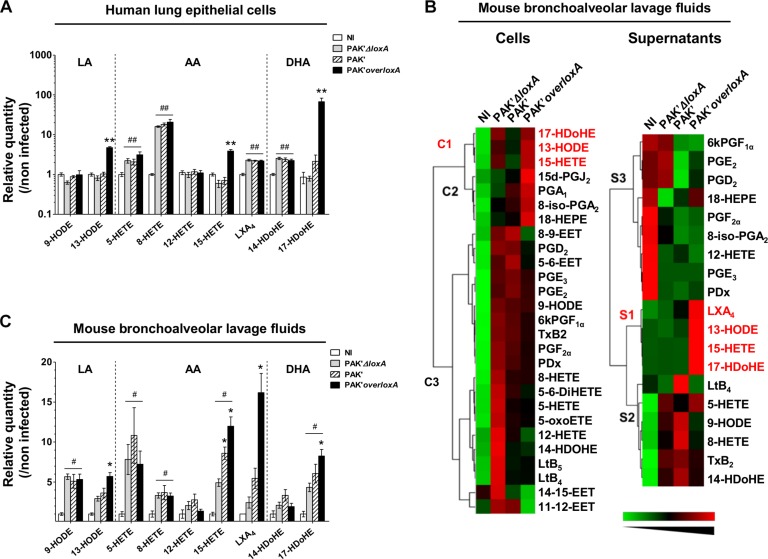
*Pseudomonas aeruginosa* LoxA secretion increases the production of 15-LOX-dependent metabolites in lung mucosa. **(A)** Human pulmonary epithelial NCI-H292 cells were infected with exponentially grown PAK’*ΔloxA*, PAK’ or PAK’*overloxA* strains for 24 h (MOI 0.1). LOX-derived eicosanoids were then measured by LC-MS/MS. For each condition, both cell and supernatant fractions were analyzed and were further pooled for representation. Values are mean ± SEM of fold-induction over non-infected (NI) NCI-H292 cells. Data are representative of three independent experiments. *^∗∗^p* < 0.01 when one compared PAK’*ΔloxA-* with PAK’*overloxA-*infected NCI-H292 cells. ^##^*p* < 0.01 when one compared PAK’*ΔloxA* or PAK’*overloxA* vs. non-infected NCI-H292 cells. **(B)** Heat-maps represent changes in the levels of 28 eicosanoid species in cells and supernatant fractions of BALF collected from PAK’*ΔloxA*- or PAK’*overloxA*-infected mice relatively to uninfected animals *(n* = 5–6 mice/group). Increase in metabolite levels are indicated in *red*, decrease in *green*, and detectable but unchanged levels in *black*. Metabolites below the limit of detection are not indicated. The name of 15-LOX-dependent by-products are written along the heat-maps in red. Lipid metabolite clusters in both cellular, and supernatant fractions are also indicated. EET, epoxy-eicosatrienoic acids; HDoHE, hydroxy-docosahexaenoic acid; HEPE, hydrox-eicosapentaenoic acid; HETE, hydroxy-eicosatetraenoic acid; HODE, hydroxy-octadecadienoic acid; Lt, leukotriene; LX, lipoxin; oxoETE, oxo-eicosatraenoic acid; PD, protectin; PG, prostaglandin; Tx, thromboxane. **(C)** Quantitative analysis of LOX-dependent metabolites. Balb/c mice were intranasally instilled with exponentially grown PAK’*ΔloxA* or *overloxA* strains for 24 h (0.5–1.10^8^ cfu/mice). At 24 h post-infection, LOX-derived eicosanoids in BALF were measured by LC-MS/MS. For each condition, both cell and supernatant fractions were analyzed and were further pooled for representation. Values are mean ± SEM of fold-induction over non-infected (NI) animals (*n* = 5–7 mice/group). ^*^p < 0.05*, ^∗∗^p* < 0.01 when one compared PAK’*ΔloxA-* with PAK’ or PAK’*overloxA-*infected mice. ^#^*p* < 0.05*, ^##^p* < 0.01 when one compared PAK’*ΔloxA* or PAK’*overloxA* vs. non-infected animals. Data are representative of three independent experiments. All absolute quantification values are available in Supplementary Tables S1, S2.

LC-MS/MS analysis indicated that *P. aeruginosa*-triggered epithelial cell infection induces the production of AA-derived 5-Hydroxyeicosatetraenoic acid (5-HETE), 8-HETE and lipoxin A_4_ (LXA_4_), DHA-derived 14-Hydroxydocosahexaenoic acid (14-HDoHE), and regardless of *loxA* expression (Figure 2A and Supplementary Table S1). By contrast, the 15-LOX-dependent metabolites 13-hydroxy-octadecadienoic acid (13-HODE), (15-HETE), and (17-HDoHE) derived from LA, AA and DHA, respectively, were increased only in epithelial cells infected by *P. aeruginosa* expressing *loxA* (fold-induction of 13-HODE: 4.7 ± 0.3 vs. 0.8 ± 0.1; 15-HETE: 3.9 ± 0.4 vs. 0.6 ± 0.1 and 17-HDoHE: 67.1 ± 15.7 vs. 0.8 ± 0.1 by the PAK’*overloxA* and PAK*’ΔloxA* strains, respectively; *p* < *0.01*). Hence, our results show a specific impact of *P. aeruginosa* LoxA on the level of 15-LOX catabolites in human lung epithelial cells. By contrast no significant difference could be observed in bacterial load (Supplementary Figure S2A) or in the secretion of 102 cytokines and chemokines induced by either PAK’*ΔloxA* or PAK’*overloxA* (Supplementary Figure S3).

### LoxA Secreted by *P. aeruginosa* Modulates the Immune Lipid Signaling in Lungs Tissues

Regulation of lipid-based inflammation is a complex process that involves lipid transcytosis and crosstalk between different cell types (e.g., epithelial and immune cells) which have distinct enzymatic repertoire (Serhan, 2007; Serhan et al., 2008). We assessed the ability of *loxA*-expressing *P. aeruginosa* strain to interfere with the host lipid signaling *in vivo* using a mouse model of acute lung infection. We challenged for 24 h mice with either PAK’ or the PAK*’ΔloxA* or PAK*’overloxA* mutant strains and compared the lipidomic profile in the cell and supernatant fractions of bronchoalveolar lavages fluids (BALF). As shown in Figure 2B, production of lipid mediators derived from CYP- COX- and LOX pathways was found to be modified by *P. aeruginosa* infection, irrespectively of *loxA* expression, in both cellular (PUFAs cluster C3) and supernatants fractions (PUFAs clusters S1 and S3). By contrast, specific clusters of 15-LOX-dependent end-products of LA, AA, and DHA metabolism was increased in BALF fractions (clusters C1 and S2) from PAK*’overloxA*-infected mice. For instance, fold-induction of some of those lipid metabolites were as follows: 13-HODE, 5.7 ± 0.5 vs. 2.9 ± 0.3; 15-HETE, 12 ± 1.17 vs. 4.9 ± 0.5; 17-HDoHE, 8.6 ± 0.8 vs. 4.3 ± 0.5 in PAK’*overloxA-* vs. PAK*’ΔloxA-*infected lungs, respectively, *p* < 0.05; Figure 2C and Supplementary Table S2). Several COX-dependent metabolites co-clusterized with these specific 15-LOX metabolites in the cellular fraction (cluster C2, Figure 2B). Moreover, the level of the potent pro-resolution lipid LXA_4_ was ∼eightfold higher in lung fluids of mice infected with the PAK’*overloxA* strain than in the PAK*’ΔloxA* samples (16.2 ± 2.4 pg/ml vs. 2.4 ± 0.7 pg/ml, respectively, *p* < *0.01*, Figure 2C and Supplementary Table S2). Remarkably, compared to PAK*’ΔloxA*, the level of 15-HETE was found to be significantly higher in PAK’-infected lungs. This finding was rather unexpected, considering the barely detectable expression of LoxA in PAK’s strain grown *in vitro* (Figures 1C–D). This suggests that *in vivo*, the lung environment may favor the expression and activity of LoxA, at a level sufficient for increasing the production of lipids mediators such as 15-HETE.

### LoxA Secreted by *P. aeruginosa* Modulates the Chemokine Production in Lung Tissues

**FIGURE 3:**
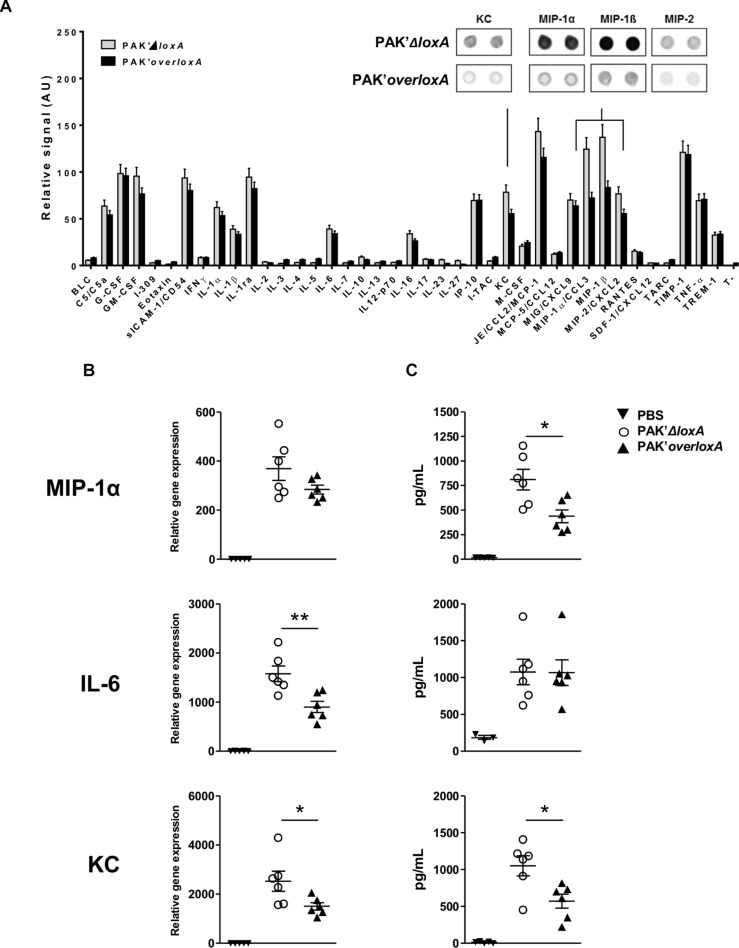
*Pseudomonas aeruginosa* LoxA secretion modulates the mucosal immune response. **(A)** Semi-quantitative representation of the levels of 40 cytokines in BALF of either PAK’*ΔloxA*-infected- or PAK’*overloxA*-infected mice, 24 h post-infection. The data were normalized to internal positive controls spotted on the same protein array membrane and are expressed as relative units. The most relevant cytokine-array spots (KC, MIP-1α, MIP-1β, and MIP-2) are shown. **(B)** Quantitative RT-PCR analysis of IL-6, KC and MIP-1α mRNA transcripts extracted from the corresponding lungs tissues. **(C)** Concentrations of IL-6, KC and MIP-1α in the same BALF as in **(A)** were determined by ELISA and are represented as the mean ± SEM (*n* = 5–6 mice/group). Data are representative of three independent experiments ^*^p < 0.05, ^∗∗^*p* < 0.01.

In order to also evaluate the impact of *loxA* expression on the production of non-lipid mediators in lung tissues, we further compared the level of 40 molecules in BALF from mice infected by either PAK*’ΔloxA* or PAK*’overloxA* strain (Figure 3A). Among those immune mediators, KC (CXCL-1) and distinct “Macrophage-Inflammatory Proteins” (i.e., MIP-1α/CCL-3, MIP-1β/CCL-4, and MIP-2/CXCL-2) were decreased in lung fluids of PAK*’overloxA*-infected animals. This was confirmed by either qRT-PCR and/or ELISA (Figures 3B,C). For instance, concentrations of both KC and MIP-1α were ∼50% less in samples collected from PAK*’overloxA* strain-infected mice). These results show that *in vivo, P. aeruginosa* LoxA down-regulates host pro-inflammatory mediators, although this was not accompanied by a decrease of lung bacterial load or animal mortality (Supplementary Figure S2B). Of note, western-blotting analysis revealed a clear band corresponding unambiguously to LoxA protein at 4 h post-infection (Supplementary Figure S7). The LoxA signal at 24 h post-infection was found barely detectable.

### Recombinant *P. aeruginosa* LoxA Alters Lipid Signaling and Immunity in Lung Mucosa

**FIGURE 4:**
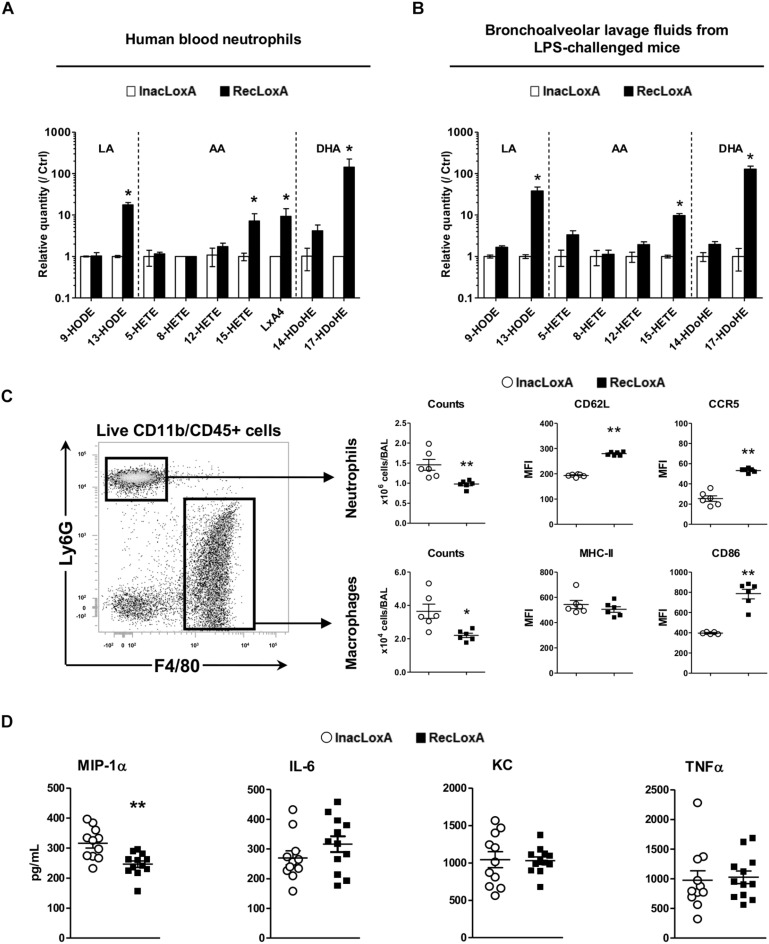
LoxA activity impacts lung mucosal defense. **(A)** Blood neutrophils from 2 independent healthy donors were incubated with either active LoxA (RecLoxA; 0.5 μg) or heat-inactived (InacLoxA; 0.5 μg) as described in the experimental procedures. For each experiments, levels of lipoxygenase-derived eicosanoids were measured by LC-MS/MS. Data are expressed as mean ± SEM of fold-induction over values measured in neutrophils incubated with InacLoxA (*n* = 3). Data are representative of two independent experiments, ^*^p < 0.05 when compared to inactive InacLoxA. See Supplementary Table S3 for absolute quantification values. **(B)** Balb/c mice were intranasally instilled with LPS (3 μg/mice) alone or with either active recombinant LoxA (RecLoxA, 5 μg/mice) or heat-inactivated recombinant LoxA (InacLoxA; 5 μg/mice; as negative control). Levels of lipoxygenase-derived eicosanoids in BALF were measured by LC-MS/MS after 24 h. For each experiment, both cell and supernatant fractions were analyzed and pooled for representation. Data are expressed as mean ± SEM of fold-induction over values measured in mice challenged by LPS and InacLoxA. See Supplementary Table S4 for absolute quantification values. Data are representative of three independent experiments, ^*^p < 0.05 vs. LPS-InacLoxA. **(C)** Gating strategy used in flow-cytometry analysis. Cell recruitment and cell-surface expression of CD62L and CCR5 on neutrophils or cell-surface expression of CD86 and MHC-II on macrophages were analyzed by flow cytometry in BALF. Data are the mean ± SEM (*n* = 6 mice/group) and are representative of three distinct experiments. ^*^p < 0.05; ^∗∗^*p* < 0.01 vs. LPS/InacLoxA. **(D)** Concentrations of IL-6, KC and MIP-1α in BALF were determined by ELISA and are represented as the mean ± SEM (*n* = 12 mice/group). ^*^p < 0.05.

To get a better insight into the specific role of LoxA activity on the host immune system, irrespectively, of any other *P. aeruginosa* virulence factor, we examined first the *ex vivo* effect of a recombinant LoxA (RecLoxA) on the lipid metabolism of neutrophils. Indeed, those leukocytes are the first to be recruited upon *P. aeruginosa* infection. As shown in Figure 4A, the level of several 15-LOX dependent by-products was increased in the presence of active RecLoxA (fold–increases are 17.6 ± 9.2 for 13-HODE, 7.2 ± 3.5 for 15-HETE, 9.3 ± 4.9 for LXA_4_, and 143.3 ± 82.2 for 17-HDoHE and Supplementary Table S3). Of note, RecLoxA did not impair killing properties of neutrophils as assessed by the measurement of proteases, NETs or ROS release (data not shown). Next, we assessed the effect of LoxA *in vivo* using mice intranasally challenged with RecLoxA and lipopolysaccharide (LPS; being used as a potent bacterial immuno-stimulus). In comparison with heat-inactivated RecLoxA (InacLoxA), RecLoxA enhanced the production of the main 15-LOX-dependent derivatives of LA, AA, and DHA in the airways (fold–change of 13-HODE: 38.1 ± 9.2; of 15-HETE: 9.8 ± 1.1; of 17-HDoHE: 128.3 ± 23.5; *p* < 0.05; Figure 4B and Supplementary Table S4). We also found a lower number of neutrophils and macrophages in mice receiving LPS and RecLoxA compared to those exposed to LPS and InacLoxA (1.46 ± 0.14 vs. 0.97 ± 0.04 × 10^6^ neutrophils, *p* < 0.01; and 3.65 ± 0.43 vs. 2.2 ± 0.13 × 10^4^ macrophages, *p* < 0.05; Figure 4C). Interestingly, neutrophils collected from the BALF of mice treated with LPS and RecLoxA expressed a higher level of CD62L (MFI: 279.8 ± 2.7 vs. 193.1 ± 2.2 in neutrophils from mice challenged with LPS and InacLoxA, respectively, *p* < 0.01; Figure 4C). As CD62L shedding is a well-known marker of neutrophil activation (Wagner and Roth, 2000), our results suggest that *P. aeruginosa* LoxA decreases the activation of this major leukocyte. Besides, we found in neutrophils exposed to LPS and RecLoxA, a significant increased expression of CCR5, a chemokine scavenger receptor (Figure 4C). Similar findings were obtained when we focused on macrophages and CD86, a co-stimulatory molecule known to be critical during the resolution phase of inflammation (Moser et al., 2014). By contrast, expression of the activation marker MHC-II was not modified, suggesting a restricted immunomodulatory effect of LoxA. Nor did we find a regulation of major inflammatory cytokines such as IL-6, KC, and TNFα in BALF of mice challenged with RecLoxA and LPS (Figure 4D). By contrast and in accordance with the results obtained with the PAK’*overloxA* strain (Figure 3), a significant decrease of the pro-inflammatory cytokine MIP-1α was measured (Figure 4D).

### Recombinant *P. aeruginosa* LoxA Impairs the Antibacterial Defense of the Lung Mucosa

**FIGURE 5:**
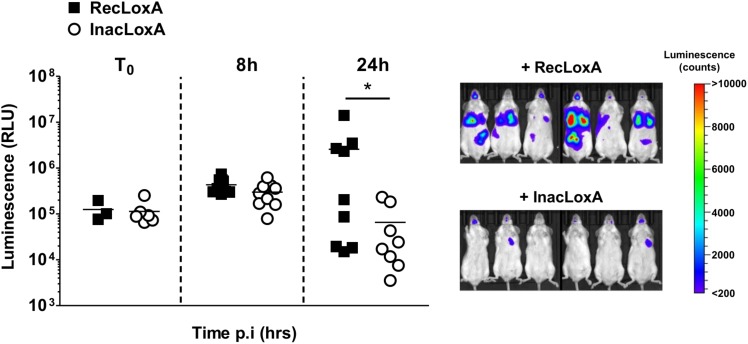
Recombinant LoxA activity promotes *P. aeruginosa* persistence in lungs. Balb/c mice were intranasally instilled with both PAK’*ΔloxA-luxCDABE* (1.10^8^ cfu/mice) and 5 μg/mice of active LoxA (RecLoxA) or heat-inactivated recombinant LoxA (InacLoxA). Lung infection was assessed by quantifying *P. aeruginosa*-associated luminescence emission in live animals. Data represent the mean ± SEM (*n* = 6–9 mice/group) and are representative of three independent experiments, ^*^p < 0.05.

To assess the overall effect of LoxA activity on *P. aeruginosa*-triggered pneumonia, we monitored by bioluminescence imaging the time-course of the infection induced by a bioluminescent PAK’Δ*loxA* (Figure 5). Before 8 h post-infection, the amount of luminescence signal in mice exposed to RecLoxA or InacLoxA was not statistically different, indicating that the initial development of the infection was similar in both groups. By contrast, at 24 h post-infection, the amount of luminescence signal in the lungs of RecLoxA-treated mice was markedly increased, suggesting a higher amount of *P. aeruginosa* (254.5 ± 151.8 vs. 5.8 ± 2.9 × 10^4^ RLU for RecLoxA-treated and InacLoxA-treated mice, respectively, *p* < 0.05). Hence, our results indicate that LoxA activity may promote *P. aeruginosa* lung pathogenesis.

## Discussion

Lipoxygenases have long been restricted to eukaryotic organisms. With a few exceptions, bacteria containing predicted lipoxygenase-encoding sequences in their genome belong to Proteobacteria and Cyanobacteria phyla and are estimated to represent less than 0.5% of all sequenced bacteria. This limited gene distribution in bacteria suggests that lipoxygenase-encoding genes might be acquired by horizontal transfer (Porta and Rocha-Sosa, 2001; Hansen et al., 2013; Horn et al., 2015; Ivanov et al., 2015; Kuhn et al., 2015).

*Pseudomonas aeruginosa* is a highly versatile bacterium. One basis for its versatility is the arsenal of enzymes that helps this pathogen to adapt to its environment. By performing a screen of lipoxygenase activity on a large panel of *P. aeruginosa* clinical isolates, we show that *loxA* gene expression is strain-dependent. *LoxA* gene is highly conserved since a nucleotide BLAST analysis performed on 2226 sequences of *P. aeruginosa* isolates collected from the *Pseudomonas* database (Winsor et al., 2011) resulted in 2224 hits sharing more than 90% identity. Based on this high degree of conservation, the absence of lipoxygenase activity that we observed in many *P. aeruginosa* strains might result from a very low promoter activity rather than from an absence of the *loxA* gene. This assumption is in agreement with the first *in vitro* biochemical study on LoxA (Vance et al., 2004) which reported a weak promoter activity in the PAK strain but no secretion of active lipoxygenase protein. Conversely, we found that the *P. aeruginosa* M56 strain exhibit both a high *loxA* promoter activity and a large secretion of active LoxA. Interestingly, insertion into the PAK strain of the region containing the predicted promoter of *loxA* from M56 strain fused to a luciferase reporter did not result in a promoter activity (Supplementary Figure S4). This latter result strongly suggests that the promoter structure of *loxA-*positive strains is not sufficient to confer the ability to express LoxA and that a more complex regulatory mechanism is likely involved. In that regard, it is of interest to note that when we screened LoxA protein and *loxA* promoter activities in *P. aeruginosa* strains under conditions appropriate for biofilm formation (i.e., static and low-temperature growth conditions), we found a clear influence of bacteria growth phase and culture conditions on *loxA* expression. Transcriptional fusion analysis as well as lipoxygenase assay have been performed using a mutant defective for biofilm formation (*ΔfliC*) which allowed us to confirm that *loxA* is preferentially expressed in biofilm conditions (Supplementary Figures S5A,B). This finding is in agreement with the work reported by Deschamps et al. (2016) in which a biofilm overproducing mutant of PAO1 (*wspF*) was used to induce *loxA* gene. Interestingly, we found that *loxA* contributes to biofilm formation in M56 clinical isolate, especially in the air-liquid-interface (ALI) pellicle formation (Supplementary Figures S5C,D). The ability to form biofilm in non-mucoid strains is based on the expression of *pel* and/or *psl* operons that encode two types of exopolysaccharide biofilm matrix (Colvin et al., 2012). Hence, differences in the type of matrix exopolysaccharides produced by PAO1 and M56 strains may explain the differences observed in the contribution of *loxA* to biofilm formation.

**FIGURE 6:**
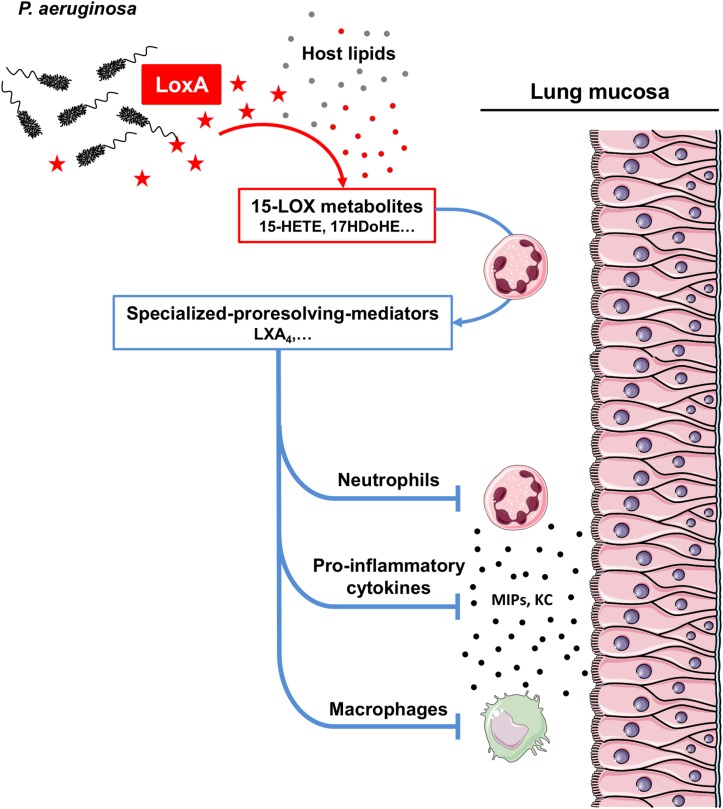
Scheme model of how LoxA contributes to *P. aeruginosa* pathogenesis. *P. aeruginosa* can naturally secrete a functional lipoxygenase LoxA (symbolized as red stars). This enzyme can process a wide range of PUFAs (shown as filled gray circles) present in the host lung mucosa, to further generate specific 15-LOX by-products (including 15-HETE, 17-HDoHE, etc; shown as filled red circles). Those metabolites can be further processed by recruited leukocytes, such as neutrophils, through a transcellular mechanism to subsequently produce specialized pro-resolving mediators (including LXA_4_). The overproduction of such highly potent lipid mediators may then downregulates the secretion of specific immune mediators (e.g., MIPs and KC) as well as the function of cells critical for the antimicrobial defense (e.g., neutrophils and macrophages). As a result, LoxA may confer a survival advantage as this enzyme reduces *P. aeruginosa* clearance in lung tissues.

Lipoxygenases belong to a large family of enzymes that metabolizes PUFAs to release bioactive lipids with potent immunomodulatory effects (Serhan et al., 2008; Tam, 2013; Dennis and Norris, 2015). Several lipoxygenase enzymatic isoforms (12-, 15-, and 5-LOX) have been described in humans, according to their carbon specificity on AA (Kuhn et al., 2015). Specific isoforms are mainly involved in the production of either pro-inflammatory (e.g., leukotrienes) or pro-resolving (e.g., lipoxins, resolvins, and maresin) mediators. Recent studies have shown that LoxA is a 15-LOX that can oxidize host AA-phosphatidylethanolamines (AA-PE) to form 15-hydroperoxy-AA-PE (Banthiya et al., 2015; Deschamps et al., 2016; Aldrovandi et al., 2018; Dar et al., 2018). In human bronchial epithelial cells, this reaction triggers ferroptosis, a cell death program (Dar et al., 2018). Here, we confirmed that *P. aeruginosa* LoxA is a 15-LOX and we extended these data by showing that this bacterial enzyme metabolizes a wide range of free host PUFAs including ω-3 fatty acids *in vitro* (Supplementary Figure S6 and Supplementary Table S7) as well as during the lung infection process *in vivo* (Figure 2). To further define a preferential free PUFA substrate of LoxA, we performed molecular modeling studies (Krammer et al., 2005), and PUFAs were classified based on scoring functions (Muegge and Martin, 1999; Muegge, 2006; Hartshorn et al., 2007). This analysis shows that DHA is the best ranked substrate of LoxA followed by EPA (Supplementary Table S7). At the molecular level, the aliphatic chain of DHA may interact with a large hydrophobic canal. The carboxylate group of DHA may directly interact with a Fe^2+^ ion and completes the coordination of the metal (Supplementary Figure S6C). As a result, LoxA-expressing *P. aeruginosa* strains trigger the production of the pro-resolving lipid LXA_4_, but not alone. Indeed, biosynthesis of LXA_4_ requires a transcellular process involving multiple cell types (e.g., endothelium, epithelium, and leukocytes) and the concerted action of at least two LOXs. Interestingly, our data using human blood neutrophils co-incubated with RecLoxA suggests that this *P. aeruginosa* enzyme produces lipid intermediates that are further metabolized by the 5-LOX present in neutrophils, to ultimately release LXA4 (Figure 6).

LoxA-dependent lipid products may further contribute to inhibit *via* an autocrine or paracrine loop, leukocyte chemotaxis, and transmigration as well as inflammatory signaling (Figure 6). Consistently, our experimental settings demonstrated that LoxA-positive *P. aeruginosa* strains could significantly down-modulate the production of several major chemokines (including KC/CXCL-1, MIP-1α/CCL-3, MIP-1β/CCL-4, and MIP-2/CXCL-2). However, such immunoregulatory effect was not associated to an increase of the bacterial burden in *P. aeruginosa-*triggered acute pneumonia. Of note is that this mouse model is characterized by a massive infiltration of innate immune cells and a robust inflammatory mucosal response. In this highly inflamed environment, the production and stability of LoxA might not be sufficient to have a noticeable impact on *P. aeruginosa* persistence. Consistent with this assumption, LoxA protein could be detected at 4 h post-infection by western-blotting analysis which revealed a clear band corresponding unambiguously to LoxA (Supplementary Figure S7). However, the LoxA signal was barely detectable at 24 h post-infection likely due to the dramatic decrease in the bacterial load as well as to an alteration of LoxA protein. Hence, we considered using an alternative model of *P. aeruginosa*-induced chronic lung infection [using agar beads; (Bayes et al., 2016)]. However, this was not possible because several strains known to trigger such chronic infection, including PAO1, and NH57388A strains did not produce LoxA activity under these experimental conditions (illustrated in Supplementary Figure S8).

Accordingly, we attempted to overcome this issue by studying lung pathogenesis in mice co-challenged by *P. aeruginosa* and RecLoxA. In this context, we confirmed that LoxA activity decreases chemokine release and we showed a lower recruitment of immune cells in the airspaces. LoxA activity was also found to modulate the cell response of macrophages and neutrophils, as assessed by the alteration of key surface receptors (i.e., CD62L, CCR5, and CD86). Regarding CCR5, this receptor is involved in MIP-1α sequestering to effectively clear this chemokine from sites of inflammation (Ariel et al., 2006; Serhan et al., 2008). Thus, the decreased level of MIP-1α in lung fluids of LoxA-exposed animals might stem from the up-regulation of CCR5 expression. More importantly, we also observed that LoxA activity promotes the spread of bacteria in lung tissues.

In conclusion, new global approaches have led to a better appreciation of both the diversity and role of eicosanoids in the regulation of host defense mechanisms (Tam, 2013; Dennis and Norris, 2015). Emerging data also show the importance of lipid signaling pathways in the control of infectious diseases (Aliberti, 2005; Haas-Stapleton et al., 2007; Russell and Schwarze, 2014; Flitter et al., 2017). Our study now reinforces this concept by showing that the lipoxygenase LoxA (i) is secreted by a significant number of clinical isolates of *P. aeruginosa* and (ii) this enzyme may contribute to the lung pathogenesis-triggered by this opportunistic pathogen.

## Materials and Methods

### Bacterial Strains, Growth Conditions, and Plasmids Construction

Bacterial strains and plasmids used in this study are listed in Supplementary Table S5. *P. aeruginosa* and *Escherichia coli* strains were grown in Luria broth (LB) at 37°C or 28°C when specified. *P. aeruginosa* biofilm formation was obtained by growing bacteria in static conditions. Gentamycin (Gm) was used at final concentrations of 30 μg.ml^–1^ for *P. aeruginosa*, and 15 μg.ml^–1^ for *E. coli*, tetracycline (Tet) was used at final concentrations of 100 μg.ml^–1^ for *P. aeruginosa*, and 10 μg.ml^–1^ for *E. coli*, ampicillin (Amp) and kanamycin (Kan) were used at final concentration of 50 μg.ml^–1^ for *E. coli*. Clinical isolates of *P. aeruginosa* have been previously described (Lanotte et al., 2004). Deletion mutants of *loxA* gene were constructed using pEX18-Gm^*R*^ vector strategy as described in Hoang et al. (1998). The vector pEX18-Gm^*R*^-Δ*loxA* was constructed by inserting a PCR overlap product containing ∼0.8 bp regions flanking the *loxA* gene using primers *loxA*_5’ (*Eco*RI) and *loxA*_int1 for the upstream region and primers *loxA*_int2 and *loxA*_3′ (*Xba*I) with indicated cloning sites described in Supplementary Table S6. The complemented *overloxA* mutants were constructed by inserting a constitutive expression cassette of the isogenic *loxA* gene into the chromosomal *att* site using pUC18T-miniTn7T derived vector as previously described by Choi and Schweizer (2006) and Damron et al. (2013). The constitutive expression *loxA* cassette was obtained by amplifying the *loxA* (PA1169) gene using specific primers RBS-Lox (*Pst*I) and STOP-Lox (*Kpn*I) with the indicated cloning sites described in Supplementary Table S6. In the resulting pUC18T-miniTn7T-P1-*loxA* vector, the *luxCDABE* operon was replaced by the *loxA* gene under the control of the P1 constitutive promoter. Transcriptional fusions were constructed by inserting the *in silico* predicted *loxA* promoter region of M56 or PAK directly upstream the *luxCDABE* reporter operon into the mini-CTX*lux* vector (Becher and Schweizer, 2000) with the indicated cloning sites described in Supplementary Table S6. This region was obtained by amplifying the 1 kpb upstream region of *loxA* (PA1169) gene with primers P*_*loxA*_*-F (*Spe*I) and P*_*loxA*_*-R (*Pst*I). All PCR fragments were obtained with Highfidelity Polymerase (Kappa), then purified and digested with the indicated restriction enzymes (FastDigest, Invitrogen) before ligation with the destination vector digested with the same enzymes. All ligation products were introduced into *E. coli* chemically competent cells (XL1, Stratagene) and transformants were selected on LB-agar plates supplemented with antibiotics. All constructions were verified by sequencing. Vectors pUC18T-miniTn7T and derived vectors were co-electroporated with the transposase encoding helper plasmid pTNS3 into *P. aeruginosa* strains. Mini-CTX1 vectors and derived vectors were co-conjugated into either PAK or M56 strains by triparental mating using the *E. coli* HB101 as helper strain. Clones with transposon insertion were selected on LB-agar plates supplemented with antibiotics. All chromosomal integration were verified by PCR using the primers listed in Supplementary Table S6.

### Lipoxygenase Assay

To detect activity in supernatants *per se*, *P. aeruginosa* strains were grown as single colonies on LB agar overnight. A single colony was then inoculated into 10 ml of LB broth and grown to stationary phase under static conditions at 28°C. The culture broth was then centrifuged at 8000 × *g* for 15 min and the supernatant collected in a syringe, sterilized with a 0.22 μm polymer filter (Corning Star Corporation, Cambridge, MA, United States), and 20 fold concentrated using Vivaspin ultrafiltration columns, 10 kDa cut-off PES membranes (Millipore) and stored at −80°C until the assay. The lipoxygenase assay has been adapted from Anthon and Barrett (2001), in this assay, the lipid hydro peroxide acts as an oxidant for the oxidative coupling of 3-methyl-2-benzothiazolinone (MBTH, Sigma-Aldrich) with 3-(dimethyl-amino)-benzoic acid (DMAB, Sigma-Aldrich) to further generate a blue colored product. Briefly, 10 μl of sample (concentrated supernatants or recombinant enzyme suspension) were mixed with 100 μl of solution A (0.5 mM purified LA, 10 mM DMAB prepared in 100 mM phosphate buffer, pH 6) and incubated for 20 min before the addition of 100 μl of supernatant from each well were transferred into a new plate, and their absorbance was measured at 598 nm. In each plate, recombinant *Pseudomonas* 42A2 lipoxygenase and *ΔloxA* mutants were included as positive and negative controls, respectively. The mean value measured in cultures of PAK*ΔloxA* mutant’s was considered as background signal and was systematically subtracted. To detect activity in supernatants *per se*, *P. aeruginosa* strains were grown overnight to form single colonies on LB agar. A single colony was then inoculated into 10 ml of LB broth and grown to stationary phase. The culture broth was subsequently centrifuged at 8000 × *g* for 15 min and the supernatant collected in a syringe, sterilized with a 0.22 μm polymer filter (Corning Star Corporation, Cambridge, MA, United States). Each sample was finally 20 fold-concentrated using Vivaspin ultrafiltration columns, 10 kDa cut-off PES membranes (Millipore) and stored at −80°C until the assay.

### Detection of LoxA by Western-Blot Analysis

Clinical isolate M56 and mutants were cultivated in liquid LB medium at 28°C in static conditions until stationary growth-phase (OD_600_∼1). Bacterial culture (10 mL) was harvested and centrifuged at 8000 × *g* for 15 min. The supernatants (9 ml) were filtered and were further 100 fold-concentrated by ultrafiltration (Vivaspin 10 kDa cut-off PES membranes). Next, samples were separated by SDS-PAGE (4–15%) and transferred onto nitrocellulose membranes for Western-blotting. A primary rabbit polyclonal antibody raised against RecLoxA was used at a 1:1,000 dilution and a secondary antibody conjugated to the horseradish peroxidase (HRP) was used at 1:10000 dilution. The membranes were developed using ECL Prime Western Blotting System (GE Helathcare) according to manufacturer instructions.

### RNA Preparation and Real-Time PCR Amplification

For bacterial RNA analysis, *P. aeruginosa* strains were grown overnight as single colonies on LB agar at 28°C. Fresh colonies were resuspended into 20 ml of LB broth (starting OD_600*nm*_ < 0.1) and grown statically up to OD_600*nm*_ ∼1 (corresponding to late exponential growth phase). Ten milliliters of culture broth were centrifuged at 16000 × *g* for 3 min. Total RNA was extracted from pellets using TRIzol^T*M*^ Max^T*M*^ Bacterial RNA Isolation Kit (Thermo Fisher Scientific, France) according to the manufacturer’s instructions. Twenty micrograms of RNA were treated with deoxyribonuclease (DNase) using the Turbo DNA-free Kit (Invitrogen) according to the manufacturer’s instructions. Total RNA from mouse lung tissues was extracted using the NucleoSpin RNA kit (Macherey-Nagel, Düren, Germany), including a step of genomic DNA digestion with DNase. Single-stranded cDNA was synthesized from 2 μg total RNA from each sample with the High Capacity cDNA reverse transcription kit (Life Technologies SAS, Saint Aubin, France). mRNA levels were determined by quantitative real-time PCR with a LightCycler 480 instrument (Roche Diagnostics, Meylan, France). The primer pairs used are described in Supplementary Table S6. PCR was carried out by using SYBR Premix Ex *Taq* (2×) (TaKaRa Bio Europe, Saint-Germain-en-Laye, France) following manufacturer instructions in either 96-well plates for bacterial mRNA analysis or 384-well plates for lung homogenates mRNA analysis. The thermal protocol included an initial denaturation step at 95°C for 30 s followed by 40 cycles of denaturation at 95°C for 5 s and primer annealing and extension at 60°C for 20 s. Melting curves were generated for each amplified cDNA to check the specificity of the reactions.

### Culture and Infection of Pulmonary Epithelial Cells

Human pulmonary epithelial NCI-H929 cells were obtained from the American Type Culture Collection (ATCC, Manassas, VA, United States). Cells were grown in RPMI 1640 Glutamax medium (Gibco, Life Technologies, Saint-Audin, France) supplemented with 10% heat-inactivated FBS (Lonza, Walkersville, United States) in a humidified incubator with 5% CO_2_ at 37°C. For infection experiments, cells were cultivated in 24-well plates until confluence (5.10^5^ cells *per* well). Exponential growth phase bacteria (LB, 37°C, 180 rpm, OD_600*nm*_∼0.5) were washed twice in ice-cold PBS before addition to freshly dispensed cell culture medium to obtain MOI = 0.1. After 20 h, supernatant cultures were harvested, and cells were gently washed twice with ice-cold PBS before a mild scraping and a resuspension in 500 μl PBS. Cell suspensions and supernatants were then centrifuged at 8000 × *g* for 10 min, collected in a syringe and sterilized with a 0.22 μm polymer filter (Corning Star Corporation, Cambridge, MA, United States) before immediate snap-freezing and storage in liquid nitrogen until lipid mediators extraction.

### Lipid Extraction and Liquid Chromatography/Tandem Mass Spectrometry (LC-MS/MS)

Samples were subjected to solid-phase extraction, with HRX-50 mg 96-well plates (Macherey Nagel, Hoerd, France) as described previously (Le Faouder et al., 2013). This method was optimized to obtain an accurate separation of 32 molecules, with a very high sensitivity of detection and analysis (0.6–155 pg): 6kPGF_1α_, TXB_2_, PGE_2_, PGD_2_, PGA_1_, 8-isoPGA_2_, PGE_3_, 11β-PGF2α, PGF2α, LXA_4_, LXB_4_, RvD_1_, RvD_2_, 7-MaR1, LTB_4_, LTB_5_, PDx, 13-HODE, 9-HODE, 18-HEPE, 5,6-DiHETE, 15-HETE, 12-HETE, 8-HETE, 5-HETE, 17-HDoHE, 14-HDoHE, 14,15-EET, 11,12-EET, 8,9-EET, 5,6-EET, and 5-oxo-ETE in cell lysates and supernatants. For simultaneous separation of the 32 lipids of interest and the three deuterated internal standards, LC-MS/MS analysis was performed on an ultra-high performance liquid chromatography system (UHPLC, Agilent LC1290 Infinity) coupled to an Agilent 6460 triple quadrupole mass spectrometer (Agilent Technologies). The limit of detection (LOD) and the limit of quantification (LOQ) were determined for the 32 compounds, from the signal-to-noise ratio (S/N). The LOD was defined as the lowest concentration giving a signal-to-noise ratio greater than 3, and the LOQ was the lowest concentration giving a signal-to-noise ratio greater than 5. Values below the LOQ were not considered. Blank samples were evaluated, and their injection yielded no interference (no peak detected), during the analysis. Hierarchical clustering and heat-map were obtained with R^1^. PUFA metabolite quantities were transformed to z-scores and clustered based on 1-Pearson correlation coefficient as distance and the Ward algorithm as agglomeration criterion.

### Cytokine Production Analysis

Cytokine array was performed on BALF with the mouse cytokine array kit from R&D Systems according to the manufacturers’ protocol. Concentration of IL-6, KC (CXCL1) MIP-1α in BALF were quantified with Duo-Set ELISA kits (R&D Systems, Lille, France), according to the manufacturers’ protocol.

### Co-incubation of Recombinant LoxA and Human Blood Neutrophils

Blood samples were collected from healthy volunteers from the Etablissement Francais du Sang of Tours (France). Neutrophils were isolated from EDTA–whole blood by negative magnetic selection using the EasySep Direct Human Neutrophil Isolation Kit (Stemcell Technologies, Cambridge, United Kingdom). Purified neutrophils, 5.10^6^ cells in 1X of 450 μl Hank’s Balanced Salt Solution (HBSS) were incubated with 50 μl of either recombinant LoxA (RecLoxA) suspension or heat-inactived LoxA (InacLoxA, incubated 10 min at 95°C) (10 μg/ml in HBSS) and gently mixed during 15 min at 25°C. Neutrophils were then stimulated by adding 5 μl of 100 μM calcium ionophore solution (A23187) and 50 μl of CaCl_2_ (50 mM) during 15 min at 37°C. Cell suspensions were then centrifuged at 300 × *g* for 10 min. Supernatants were further mixed with 300 μl of pure ice-cold methanol and pellets were resuspended in 200 μl of PBS before snap-freezing in liquid nitrogen for storage.

### Mouse Challenge by LPS and Infection by *P. aeruginos*a

Balb/c mice (males) were used at ∼8 weeks of age and supplied by Janvier Laboratories (Le Genet Saint Isle, France). Mice were anesthetized by intra-peritoneal injection of a mixture of ketamine-xylazine and were placed supine. For lung infection experiments, each specified strains of *P. aeruginosa* was grown overnight in Luria-Bertani broth and further transferred into fresh medium and grown for 4–5 h to mid-log phase. The bacteria cultures were centrifuged at 4000 × *g* for 15 min and the cell pellets washed twice with PBS. The bacterial pellet was diluted in its original volume and the OD adjusted to give the desired inoculum. A 50-μl bacterial suspension (1.10^8^ cfu/mice) was administrated by intranasal instillation and mice were then immediately held upright to facilitate bacterial inhalation until normal breathing resumed. The inoculum was verified by serial 10-fold dilutions of the bacterial suspensions and plating on LB agar plates. For the LPS challenge experiments, a 50 μl PBS suspension containing 3 μg of *Escherichia coli* O111:B4 LPS (Sigma-Aldrich, L2034) was administrated by the intranasal route, concomitantly with 5 μg of either active or heat-inactivated (InacLoxA, 10 min 95°C) recombinant *Pseudomonas aeruginosa* 42A2 lipoxygenase (provided by X. Carpena). BALF were performed 24 h after instillation, after a pentobarbital euthanasia procedure.

### *In vivo* Measurement of *P. aeruginosa*-Associated Luminescence

Photon emission of luminescent *P. aeruginosa* (PAK’Δ*loxA-luxCDABE*) in the mouse was measured using the IVIS Lumina XR system (Perkin Elmer), which includes an IVIS charge-coupled device camera coupled to the LivingImage software package (Perkin Elmer). Analysis of photons was done under isoflurane inhalation anesthesia. A digital false-color photon emission image of the mouse was generated, and photons were counted using a 3-min acquisition time using the following settings: Medium binning (M), Field Of View: 12.5 cm (D), f1. Image analysis and luminescence quantification have been performed with LivingImage software (Perkin Elmer). Region Of Interest (ROI) were defined as area corresponding to the surface of the chest encompassing the whole lung region after 3 min acquisition time.

### Flow Cytometry Analysis

Flow cytometry experiments were performed using a MACSQuant^®^ Analyzer (Miltenyi Biotec) and analyzed using VenturiOne software (AppliedCytometry). The following mAbs were used: FITC-conjugated anti-CD62L (MEL-14), APC-conjugated anti-Ly6G (1A8), PE-Cy7-conjugated anti-CD11b (M1/70), PE-conjugated anti-CD195 (CCR5) (C34-3448) from BD biosciences (East Rutherford, NJ, United States). Vioblue-conjugated anti-F4/80 (clone BM8), APC-eFluor780-conjugated anti-CD45 (30-F11), FITC-conjugated anti-CD86 (GL1), and PerCP-eFLuor710-conjugated anti-MHC2 (M5/114) were from Affymetrix eBioscience (Santa Clara, CA, United States). Neutrophils and macrophages were identified as CD45^+^ CD11b^+^ Ly6G^+^ cells and CD45^+^, CD11b^+^, and F4/80^+^ cells respectively.

### Quantification of Biofilm Formation

Overnight LB culture of *P. aeruginosa* was diluted in LB medium (initial OD_600_∼0.05) and dispensed into 96-well microplates (100 μL *per* well). Plates were then covered and incubated with agitation (100 rpm) in a humid chamber at either 37°C or 28°C for 48 h. Planktonic bacteria were removed by aspiration and wells were rinsed twice with PBS. The biofilms attached to wells were stained by dispensing 150 μL of crystal-violet (CV) solution (0.5% w/v) *per* well for 20 min. Unattached CV was removed by rinsing wells twice with 150 μL of distilled water *per* well. The stained material was then solubilized by incubation in 200 μL of 4:1 mixture of ethanol:acetone for 15 min. We further used 100 μL of the resulting solution for absorption measurement at 560 nm (using a Tecan M200 luminometer/spectrophotometer).

### Pellicle Formation Assay

Pellicle formation at the air-liquid interface (ALI) was visualized as previously described (Friedman and Kolter, 2004). Briefly, a standing bacteria culture containing 3-mL LB broth (OD_600_∼0.05) was grown at 28°C in a glass tube. Pellicles were visualized between 2 and 4 days later. Complete coverage at the ALI of an opaque layer of cells is considered to be indicative of pellicle formation. Planktonic cells were removed by gentle aspiration from the bottom culture and remaining bacteria were rinsed twice gently with PBS.

### Molecular Docking

LoxA was previously partially co-crystallized in presence of ZPE ((2R)-3-{[(S)-(2-aminoethoxy) (hydroxy)-phosphoryl]oxy}-2-(tetradec-5-enoyloxy)propyl (11Z)-octadec-11-enoate) and Fe^2+^(pdb code: 4G33). The structure was cleaned and co-crystallized small molecules removed. Ligands were then cleaned and prepared (i.e., protonate, tautomers, isomers, and conformer were generated) before being docked into the binding site using a protein rigid – flexible ligand protocol (cDocker). All calculations were performed in Discovery Studio 4.1. LOXA structure (PDB code: 4G33) was prepared by the use of the Prepare Protein protocol of DS 4.1 including the cleaning of the protein, the optimization of side-chain conformation for residues with inserted atoms, the removal of water molecules present in the PDB structure, the modeling of missing loop regions based on SEQRES information, and the prediction of titration site pKs and protonation state of the structure at the specified pH. Flexible ligand/rigid protein docking was performed using CDOCKER (Wu et al., 2003). Ligands were prepared using the Prepared Ligand protocol of DS 4.1 including the generation of canonical tautomers, keeping only largest fragments, the set of standard formal charges of common functional groups, the generation of Kekulé structures, enumeration of ionization states at a given pH range, enumeration of tautomers and the generation of a reasonable 3D conformation using Catalyst. Random ligand conformations were generated from the initial ligand structure through high-temperature molecular dynamics. Due to the high flexibility of ligands, we docked for each ligand several conformations previously generated with the BEST algorithm (Kirchmair et al., 2005) to cover the full range of conformers. The poses showing the lowest energy were retained and clustered according to their binding mode. Three-dimensional snapshots of the docked ligands were generated using Accelrys DS Visualizer.

### Ethics Statement

This study was carried out in accordance with the guidelines of the European Union for the care and use of animals in research protocols. This study was approved by the Comité d’Ethique en Expérimentation Animale Val de Loire (CEEAVdL) (Université de Tours, France, project license N°C 2012-12-7). All mice were housed under a reverse light–dark cycle, under standard conditions, with food and water available *ad libitum*.

### Statistics

Analyses were carried out with GraphPad Prism 5 software. Data are expressed as the mean ± SEM. We used the D’Agostino and Pearson omnibus normality test to determine whether the data were normally distributed. Differences between groups were assessed for statistical significance using the Kruskal-Wallis ANOVA test, followed by the Mann-Whitney *U* test. A value of *p* < 0.05 was considered statistically significant.

## Data Availability

The datasets generated for this study are available on request to the corresponding author.

## Author Contributions

EM, NC, XC, MS-T, RR, PL, and VH designed the experiments. EM, NC, TP-B, CB, TB, AG, DB, and NP performed the experiments. EM and MS-T wrote the manuscript.

## Conflict of Interest Statement

The authors declare that the research was conducted in the absence of any commercial or financial relationships that could be construed as a potential conflict of interest.

## Footnotes

1 http://www.r-project.org

## Supplementary Material

FIGURE S1 Influence of temperature growth conditions on LoxA production. **(A)** Upper panel: Western blot and lipoxygenase activity analysis of LoxA-positive *P. aeruginosa* M56 clinical isolate (*wt*), grown at 28 or 37°C. Mutants M56*ΔloxA* and M56*overloxA* were used as negative and positive controls, respectively. Protein supernatants extracts from stationary growth phase cultures of M56 were separated by SDS-PAGE and transferred onto nitrocellulose membranes for western-blotting using a rabbit anti-LoxA antibody. *Lower panel:* LoxA protein detection was correlated to LoxA activity by an enzymatic assay, using the same protein extracts. **(B)** Dynamic LoxA promoter activity and growth of M56-P*_*loxA*_*-*lux* grown at either 28 or 37°C, by luminescence and absorbance measurements, respectively. Colonies isolated from fresh LB-agar plates were inoculated into 4mL of LB medium (initial OD_600nm_ 0.1) into 6-well plates. Optical densities and luminescence emission were monitored for 48 h at 28°C using a Tecan M200 luminometer/spectrophotometer. **(C)** qRT-PCR analysis of *loxA* mRNA extracted from M56 static cultures grown at either 28 or 37°C and sampled at different growth phase (“early” or “late”), exponential (“exp.”) or stationary (“stat.”). All results were normalized to the signal associated to the “early exponential” growth phase condition, taken as reference.

FIGURE S2 Bacterial load in samples used for lipidomics and cytokines analysis. Bacterial load (expressed in CFU/ml) was determined at the indicated time point by plating serial dilution and further manual counting, from the following samples: **(A)** supernatants of human pulmonary epithelial NCI-H292 cells infected with exponentially grown PAK’*ΔloxA*, PAK’ or PAK’*overloxA* strains (MOI 0.1); **(B)** BALF of Balb/c mice challenged intranasally with either PAK’*ΔloxA*, or PAK’ or PAK’*overloxA* strains (1.10^8^ cfu/mice). Data represent the mean ± SEM (*n* = 5–7 mice/group) and are representative of three independent experiments.

FIGURE S3 Semi-quantitative analysis of cytokines secreted by human lung epithelial NCI-H292 cells infected by PAK’*ΔloxA* or PAK’*overloxA.* Cells were infected with exponentially grown PAK’*ΔloxA* or *overloxA* strains for 24h (MOI 0.1). Then, supernatants were centrifuged and analyzed by a cytokine-array. Data were normalized to internal positive controls spotted on the same protein array membrane and are expressed as relative units.

FIGURE S4 Dynamic *loxA* promoter activity depending on promoter sequence origin and strain expression context. The M56 clinical isolate containing *loxA* promoter sequence from PAK strain fused to *luxCDABE* reporter system (M56-P*_*loxA(PAK)*_*-*lux*) on one hand and PAK strain containing *loxA* promoter sequence from M56 strain fused to *luxCDABE* reporter system (PAK-P*_*loxA(M*56)_*-*lux*) on the other hand were grown in liquid culture and monitored for luminescence and absorbance measurements. Colonies isolated from fresh LB-agar plates were further inoculated into 4 mL of LB medium (initial OD_600 nm_ 0.1). Optical densities and luminescence emission were monitored during 48 h at 28°C in static conditions using a Tecan M200 luminometer/spectrophotometer.

FIGURE S5 The *loxA* gene is preferentially expressed in biofilm growth conditions and contributes to biofilm formation in M56 clinical isolate. **(A)** Comparison by transcriptional fusion analysis of LoxA expression in M56 clinical isolate grown in planktonic or biofilm conditions. Colonies isolated from fresh LB-agar plates were inoculated into 3 mL of LB medium (initial OD_600 nm_ 0.1) into 6-well plates and incubated at 28°C in either static conditions (“Biofilm”) or under a 130 rpm agitation (“Planktonic”) for 48 h. Cultures were then harvested, homogenized and luminescence emission was measured using the IVIS Lumina XR system (Perkin Elmer). Concomitantly, optical densities and luminescence emission quantification were analyzed using a Tecan M200 luminometer/spectrophotometer. **(B)** Comparison of LoxA activity in wild-type and biofilm-deficient M56 strain. M56 and M56Δ*fliC* mutant were cultivated in biofilm formation conditions and analyzed for biofilm formation and lipoxygenase activity. **(C)** Influence of *loxA* gene expression on biofilm formation of M56 clinical isolate and M56-derived *loxA* mutants, as assessed by CV assay. ^∗∗∗^*p* < 0.005. **(D)** Visualization of air-liquid-interface pellicle produced by M56 wild-type strain, M56Δ*loxA* and M56*overloxA* mutants.

FIGURE S6 *Pseudomonas aeruginosa* LoxA metabolizes free PUFAs to generate 15-LOX-dependent products *in vitro*. **(A)** Colorimetric detection of lipoxygenase activity depending on the PUFA used as a substrate. Recombinant LoxA (RecLoxA, 100 ng) was incubated with either linoleic acid (LA), linolenic acid (LNA), arachidonic acid (AA), eicosapentaenoic acid (EPA) or docosahexaenoic acid (DHA), as described in the experimental procedure. **(B)** right panels, show LC-MS/MS quantification of lipid metabolites after a 10 min co-incubation of purified AA or DHA (1 mM) with the indicated amounts of RecLoxA or heat-inactivated LoxA (InacLoxA). ^*^ means *p* < 0.05, vs. heat-inactivated recLoxA. **(C)** *In silico* docking of DHA to *P. aeruginosa* 42A2 LoxA. The global positioning of DHA into LoxA structure is represented in pink. The N-terminal (Nter) and the C-terminal (Cter) parts of LoxA were colored in yellow and red, respectively. The Fe^2+^ ion is represented by the blue sphere. The hydrophobic canal is represented by the brown wire mesh surface.

FIGURE S7 Detection of LoxA protein in lung tissues. Proteins were extracted from whole lung tissues of mice infected or not by PAK’*ΔloxA*, PAK’ or PAK’*overloxA* strains. Samples were obtained at 4 or 24 h post-infection and were separated by SDS-PAGE and transferred onto nitrocellulose membranes for western-blotting using an anti-LoxA antibody and an β-actin antibody (used as an internal loading control).

FIGURE S8 Comparison of lipoxygenase activity detected in reference strains (PAK and PAO1) and clinical strains (M56 and NH57388A) cultivated in planktonic or biofilm growth conditions. Colonies isolated from fresh LB-agar plates were inoculated into 3 mL of LB medium (initial OD_600 nm_ 0.1) into 6-well plates and incubated at 28°C in either static conditions (“Biofilm”) or under a 130 rpm agitation (“Planktonic”) until late stationary growth-phase. Cultures were then harvested, homogenized and analyzed for lipoxygenase activity by colorimetric lipoxygenase assay, as specified in the section “Materials and Methods.”

TABLE S1 Concentration of metabolites (pg/mg of protein) in human lung epithelial NCI-H292 cells infected or not with PAK’*ΔloxA*, or PAK’*overloxA*, at 24 h post-infection.

TABLE S2 Concentration of metabolites (pg/mg of protein) detected in BALF from mice infected or not with PAK’*ΔloxA*, or PAK’*overloxA*, at 24 h post-infection.

TABLE S3 Concentration of lipoxygenase by-products (pg/mg of protein) after incubating and activating human blood neutrophils (5.10^6^ cells) with heat-inactivated or active recombinant lipoxygenase (0.5 μg) from *P. aeruginosa* strain PA42A2.

TABLE S4 Concentration of metabolites (pg/mg of protein) in BALF from LPS (3 μg/mouse) treated mice co-challenged or not either with heat-inactivated or active recombinant PA42A2 lipoxygenase (5 μg/mouse), 24 h post-challenge.

TABLE S5 Strains and plasmids used in this study.

TABLE S6 Primers used in this study.

TABLE S7 Scoring and ranking of LoxA substrates.
