# Supplementary figures and images for: Pseudomonas aeruginosa Lipoxygenase LoxA Contributes to Lung Infection by Altering the Host Immune Lipid Signaling
Source: Front Microbiol. 2019 Aug 14;10:1826. doi: 10.3389/fmicb.2019.01826 (PMC6702342; doi:10.3389/fmicb.2019.01826)

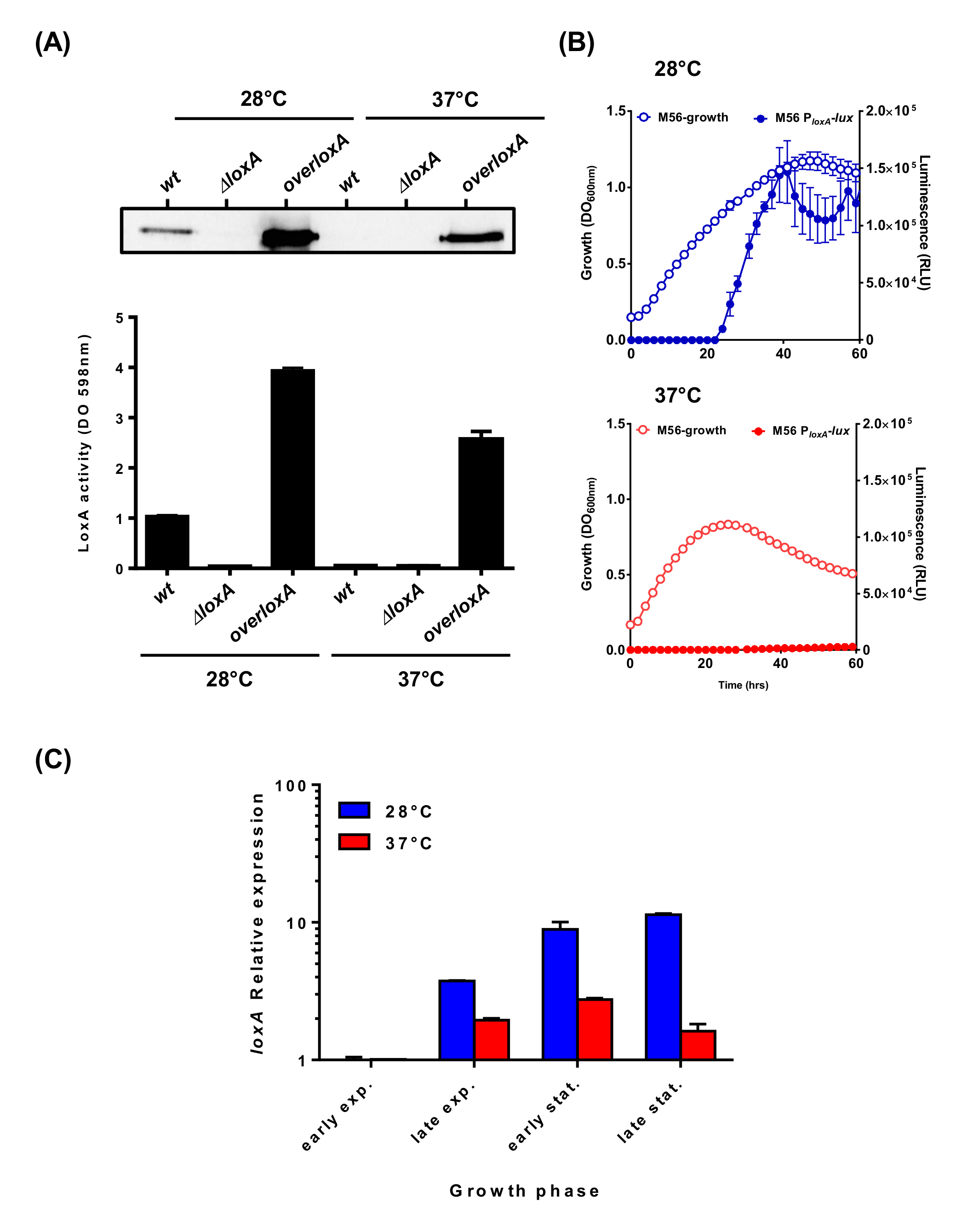

Supplement: FIGURE S1 — Influence of temperature growth conditions on LoxA production. (A) Upper panel: Western blot and lipoxygenase activity analysis of LoxA-positive P. aeruginosa M56 clinical isolate (wt), grown at 28 or 37°C. Mutants M56ΔloxA and M56overloxA were used as negative and positive controls, respectively. Protein supernatants extracts from stationary growth phase cultures of M56 were separated by SDS-PAGE and transferred onto nitrocellulose membranes for western-blotting using a rabbit anti-LoxA antibody. Lower panel: LoxA protein detection was correlated to LoxA activity by an enzymatic assay, using the same protein extracts. (B) Dynamic LoxA promoter activity and growth of M56-PloxA-lux grown at either 28 or 37°C, by luminescence and absorbance measurements, respectively. Colonies isolated from fresh LB-agar plates were inoculated into 4mL of LB medium (initial OD600nm 0.1) into 6-well plates. Optical densities and luminescence emission were monitored for 48 h at 28°C using a Tecan M200 luminometer/spectrophotometer. (C) qRT-PCR analysis of loxA mRNA extracted from M56 static cultures grown at either 28 or 37°C and sampled at different growth phase (“early” or “late”), exponential (“exp.”) or stationary (“stat.”). All results were normalized to the signal associated to the “early exponential” growth phase condition, taken as reference. [file Image_1.TIFF]

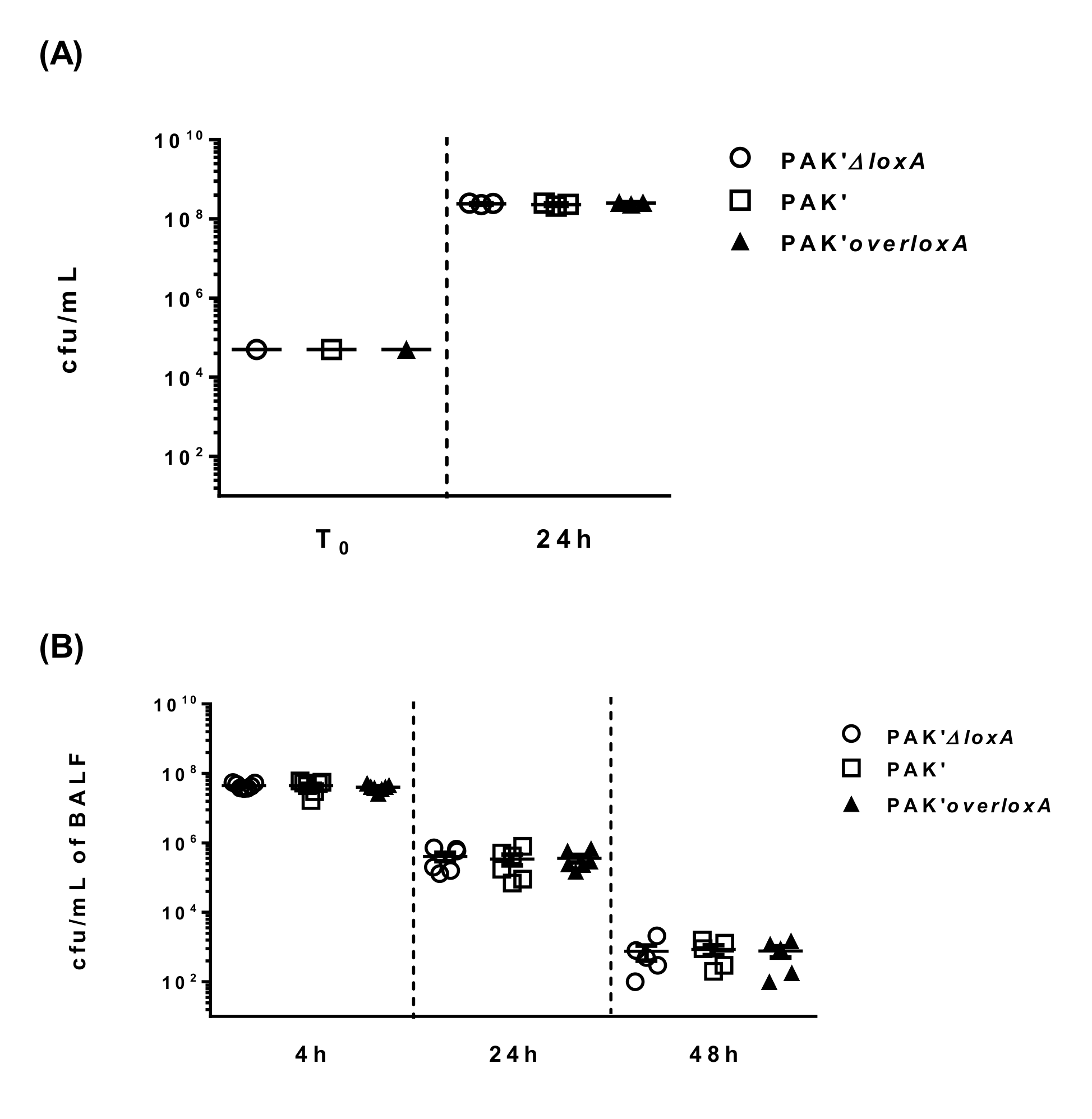

Supplement: FIGURE S2 — Bacterial load in samples used for lipidomics and cytokines analysis. Bacterial load (expressed in CFU/ml) was determined at the indicated time point by plating serial dilution and further manual counting, from the following samples: (A) supernatants of human pulmonary epithelial NCI-H292 cells infected with exponentially grown PAK’ΔloxA, PAK’ or PAK’overloxA strains (MOI 0.1); (B) BALF of Balb/c mice challenged intranasally with either PAK’ΔloxA, or PAK’ or PAK’overloxA strains (1.108 cfu/mice). Data represent the mean ± SEM (n = 5–7 mice/group) and are representative of three independent experiments. [file Image_2.TIFF]

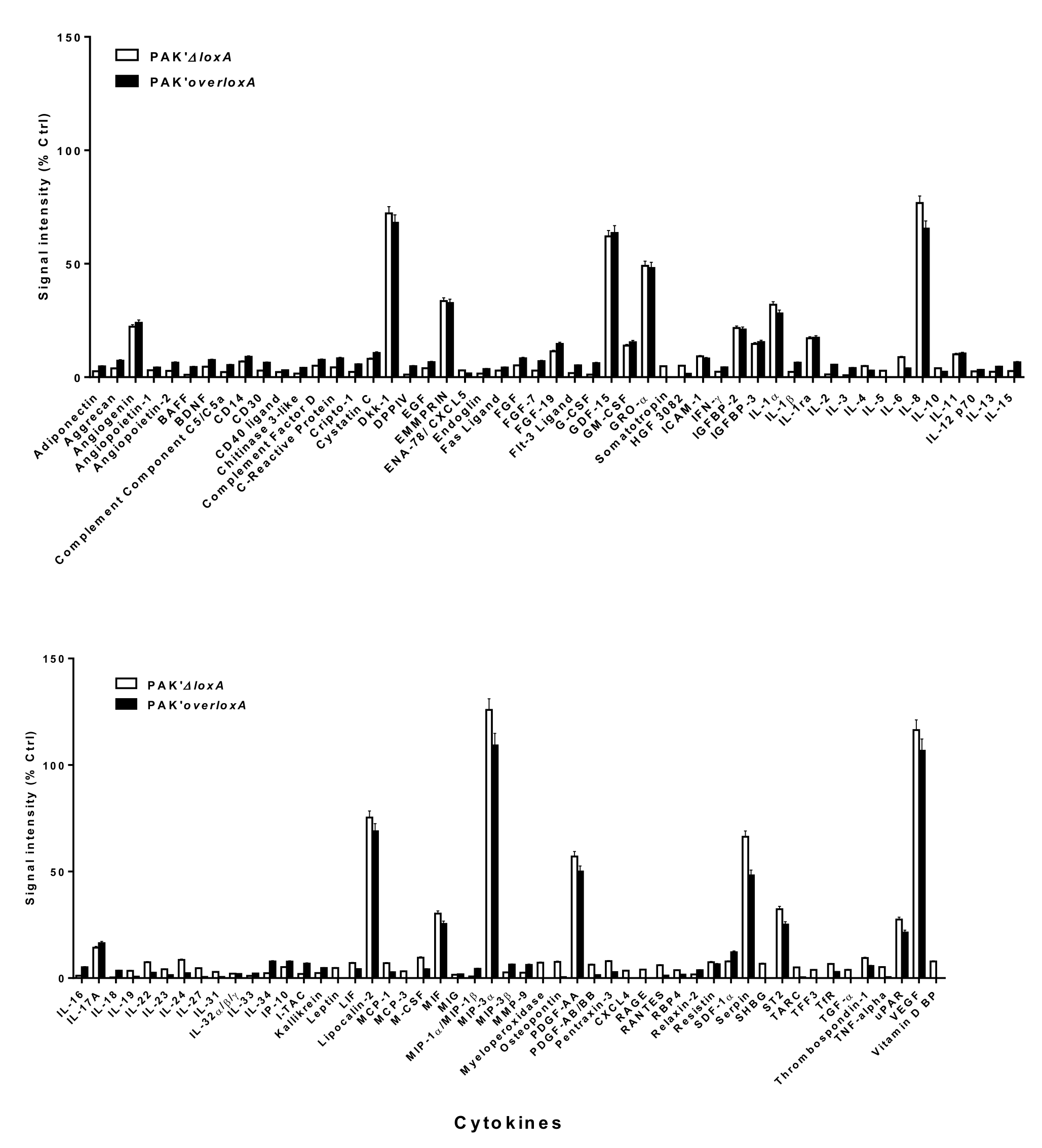

Supplement: FIGURE S3 — Semi-quantitative analysis of cytokines secreted by human lung epithelial NCI-H292 cells infected by PAK’ΔloxA or PAK’overloxA. Cells were infected with exponentially grown PAK’ΔloxA or overloxA strains for 24h (MOI 0.1). Then, supernatants were centrifuged and analyzed by a cytokine-array. Data were normalized to internal positive controls spotted on the same protein array membrane and are expressed as relative units. [file Image_3.TIFF]

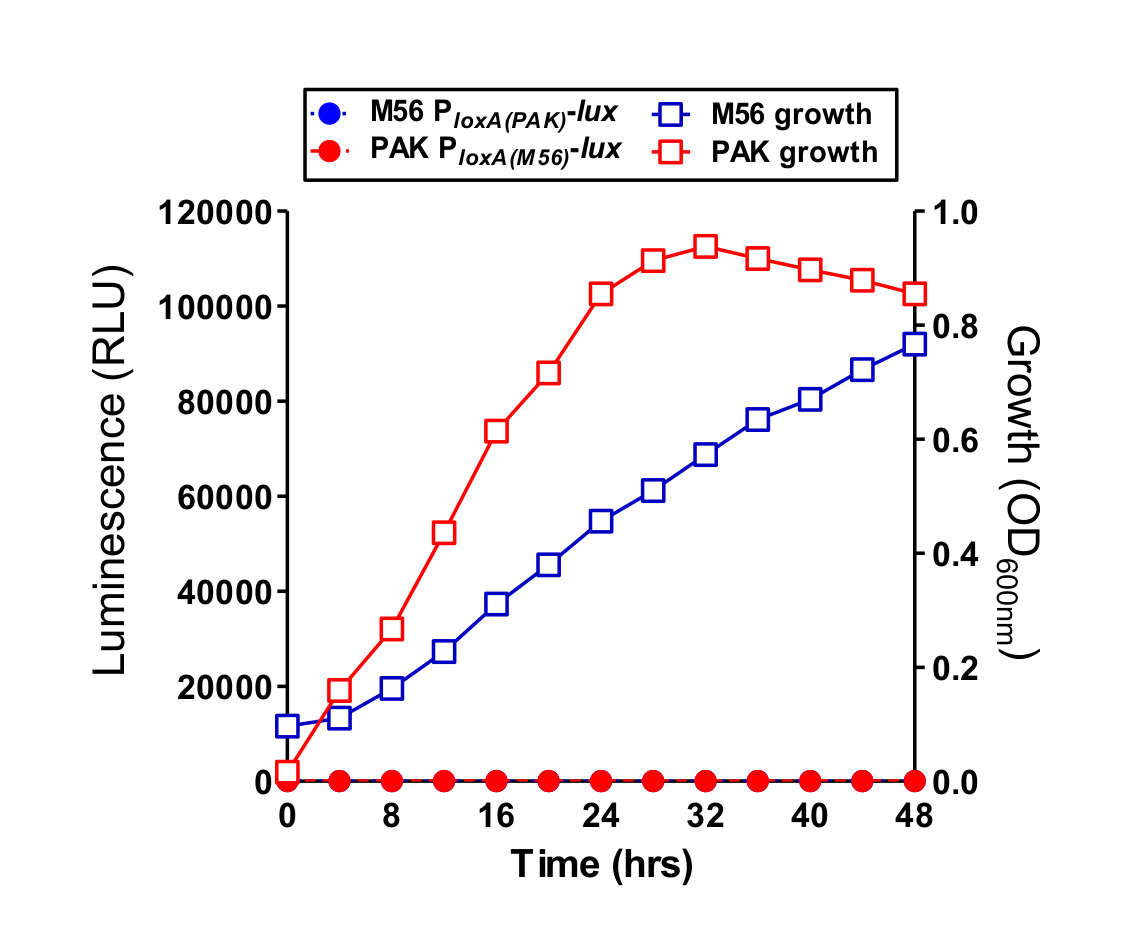

Supplement: FIGURE S4 — Dynamic loxA promoter activity depending on promoter sequence origin and strain expression context. The M56 clinical isolate containing loxA promoter sequence from PAK strain fused to luxCDABE reporter system (M56-PloxA(PAK)-lux) on one hand and PAK strain containing loxA promoter sequence from M56 strain fused to luxCDABE reporter system (PAK-PloxA(M56)-lux) on the other hand were grown in liquid culture and monitored for luminescence and absorbance measurements. Colonies isolated from fresh LB-agar plates were further inoculated into 4 mL of LB medium (initial OD600 nm 0.1). Optical densities and luminescence emission were monitored during 48 h at 28°C in static conditions using a Tecan M200 luminometer/spectrophotometer. [file Image_4.TIFF]

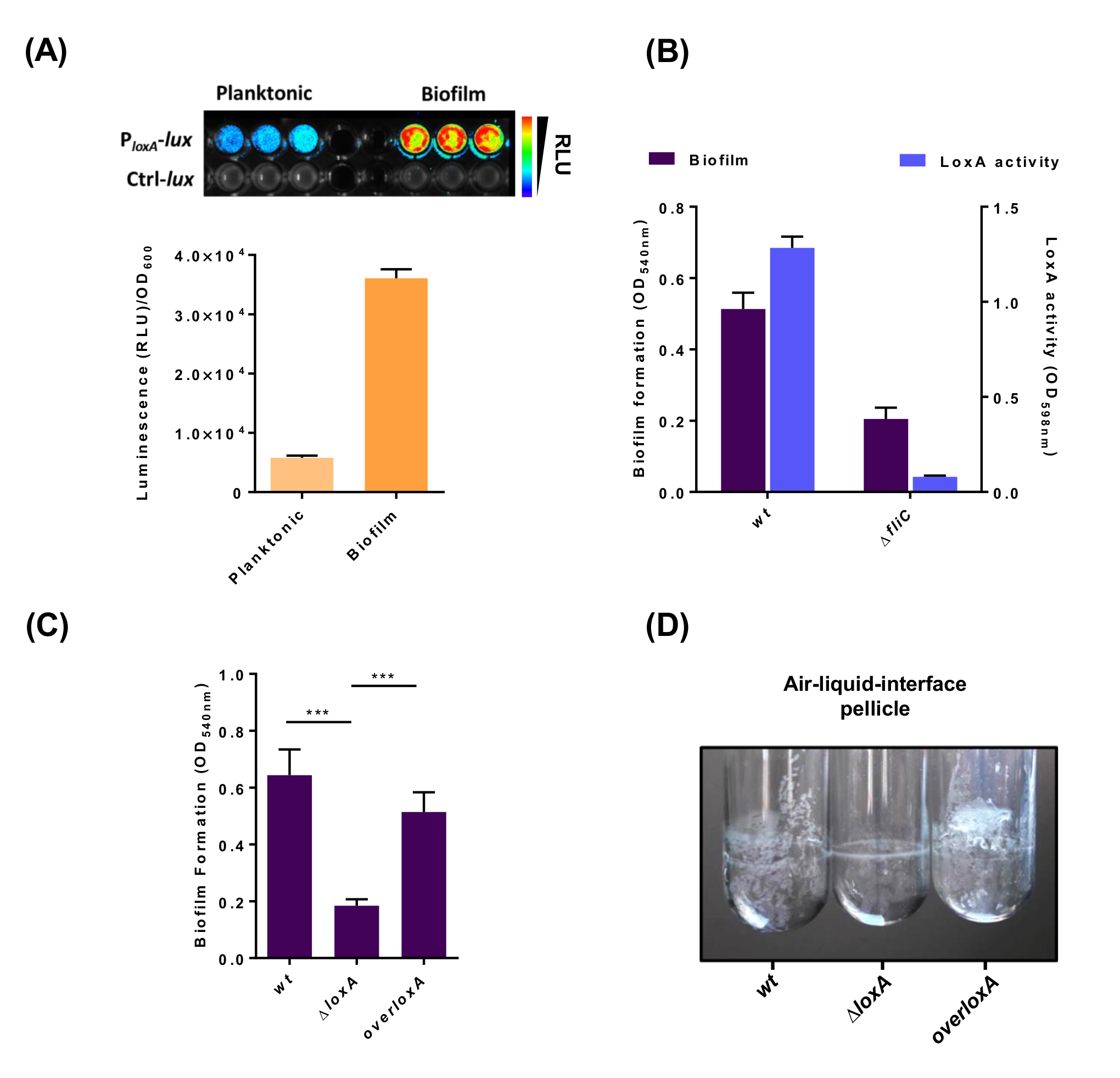

Supplement: FIGURE S5 — The loxA gene is preferentially expressed in biofilm growth conditions and contributes to biofilm formation in M56 clinical isolate. (A) Comparison by transcriptional fusion analysis of LoxA expression in M56 clinical isolate grown in planktonic or biofilm conditions. Colonies isolated from fresh LB-agar plates were inoculated into 3 mL of LB medium (initial OD600 nm 0.1) into 6-well plates and incubated at 28°C in either static conditions (“Biofilm”) or under a 130 rpm agitation (“Planktonic”) for 48 h. Cultures were then harvested, homogenized and luminescence emission was measured using the IVIS Lumina XR system (Perkin Elmer). Concomitantly, optical densities and luminescence emission quantification were analyzed using a Tecan M200 luminometer/spectrophotometer. (B) Comparison of LoxA activity in wild-type and biofilm-deficient M56 strain. M56 and M56ΔfliC mutant were cultivated in biofilm formation conditions and analyzed for biofilm formation and lipoxygenase activity. (C) Influence of loxA gene expression on biofilm formation of M56 clinical isolate and M56-derived loxA mutants, as assessed by CV assay. ∗∗∗p < 0.005. (D) Visualization of air-liquid-interface pellicle produced by M56 wild-type strain, M56ΔloxA and M56overloxA mutants. [file Image_5.TIFF]

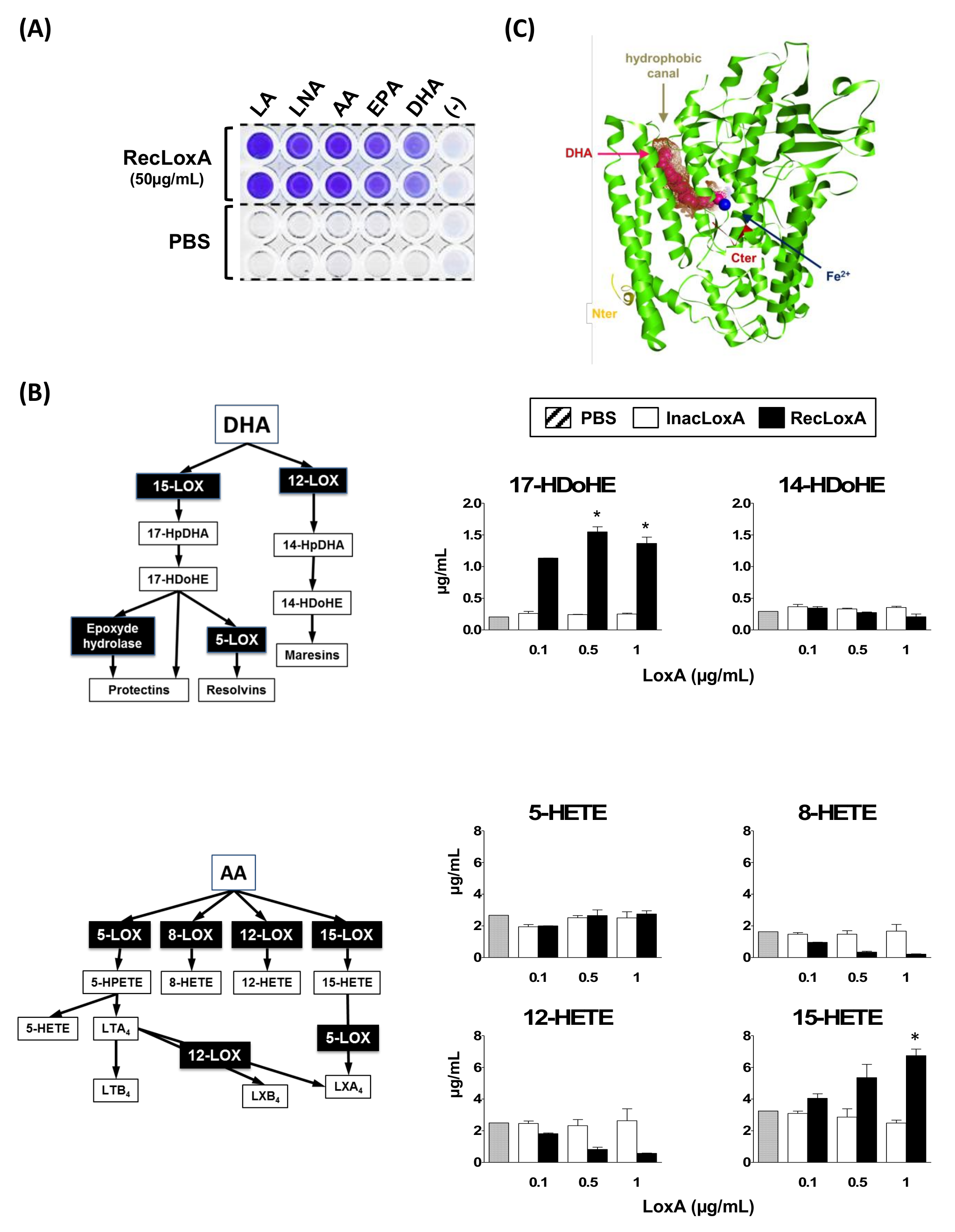

Supplement: FIGURE S6 — Pseudomonas aeruginosa LoxA metabolizes free PUFAs to generate 15-LOX-dependent products in vitro. (A) Colorimetric detection of lipoxygenase activity depending on the PUFA used as a substrate. Recombinant LoxA (RecLoxA, 100 ng) was incubated with either linoleic acid (LA), linolenic acid (LNA), arachidonic acid (AA), eicosapentaenoic acid (EPA) or docosahexaenoic acid (DHA), as described in the experimental procedure. (B) right panels, show LC-MS/MS quantification of lipid metabolites after a 10 min co-incubation of purified AA or DHA (1 mM) with the indicated amounts of RecLoxA or heat-inactivated LoxA (InacLoxA). * means p < 0.05, vs. heat-inactivated recLoxA. (C) In silico docking of DHA to P. aeruginosa 42A2 LoxA. The global positioning of DHA into LoxA structure is represented in pink. The N-terminal (Nter) and the C-terminal (Cter) parts of LoxA were colored in yellow and red, respectively. The Fe2+ ion is represented by the blue sphere. The hydrophobic canal is represented by the brown wire mesh surface. [file Image_6.TIFF]

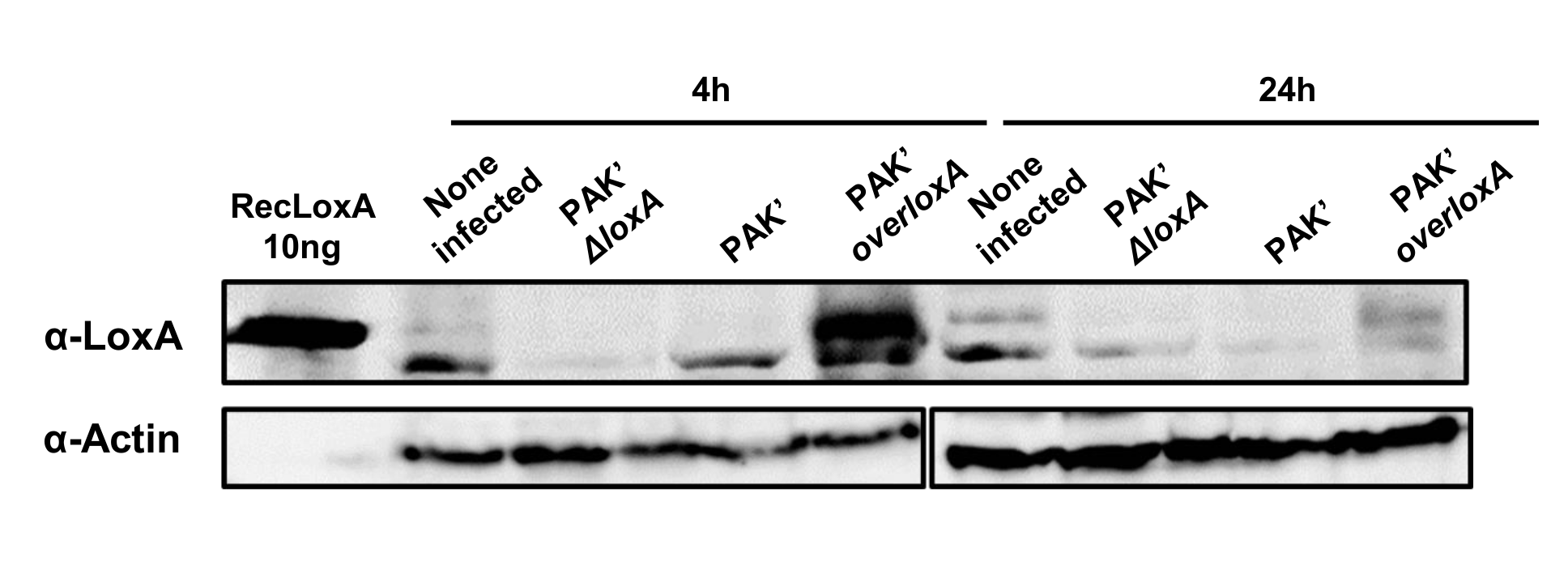

Supplement: FIGURE S7 — Detection of LoxA protein in lung tissues. Proteins were extracted from whole lung tissues of mice infected or not by PAK’ΔloxA, PAK’ or PAK’overloxA strains. Samples were obtained at 4 or 24 h post-infection and were separated by SDS-PAGE and transferred onto nitrocellulose membranes for western-blotting using an anti-LoxA antibody and an β-actin antibody (used as an internal loading control). [file Image_7.TIFF]

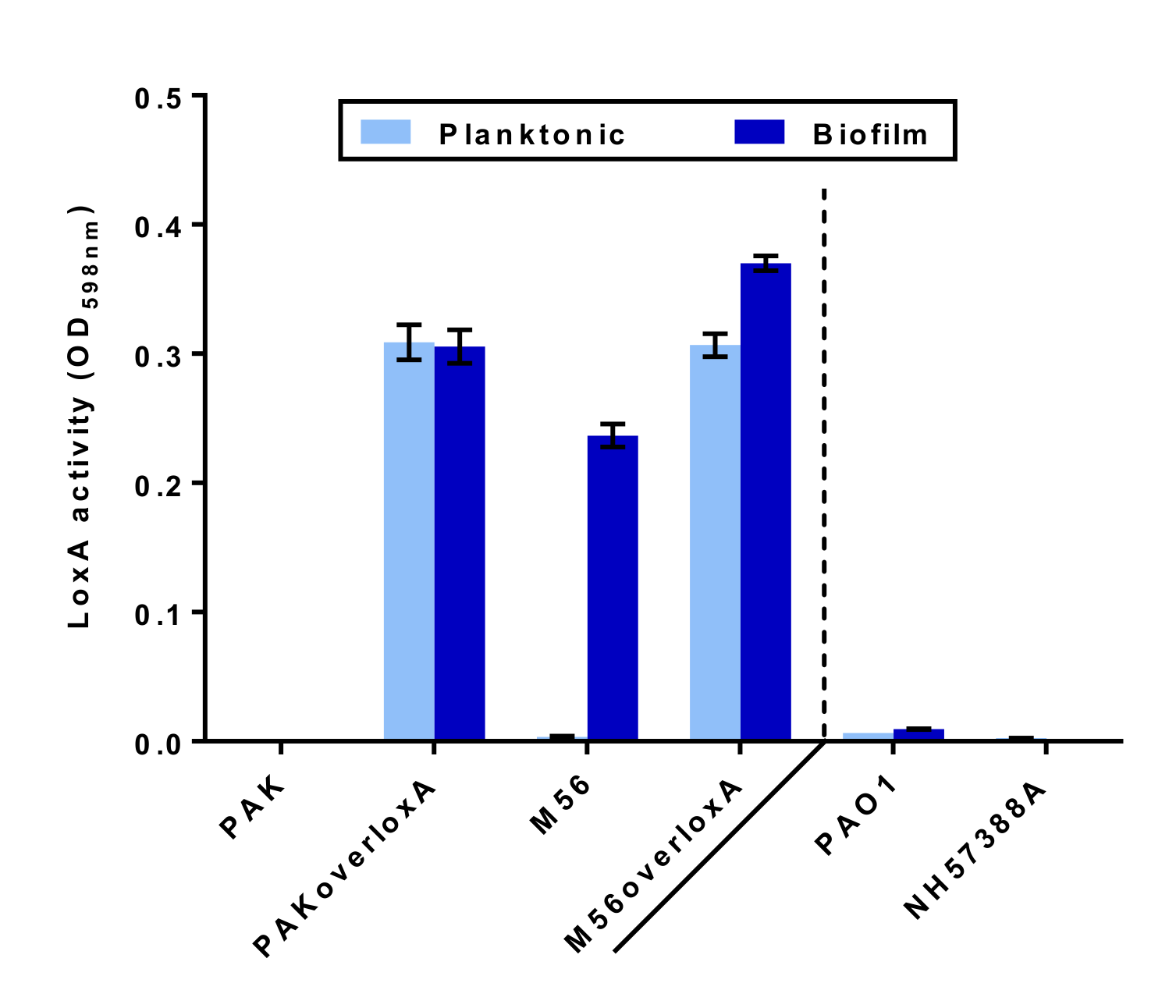

Supplement: FIGURE S8 — Comparison of lipoxygenase activity detected in reference strains (PAK and PAO1) and clinical strains (M56 and NH57388A) cultivated in planktonic or biofilm growth conditions. Colonies isolated from fresh LB-agar plates were inoculated into 3 mL of LB medium (initial OD600 nm 0.1) into 6-well plates and incubated at 28°C in either static conditions (“Biofilm”) or under a 130 rpm agitation (“Planktonic”) until late stationary growth-phase. Cultures were then harvested, homogenized and analyzed for lipoxygenase activity by colorimetric lipoxygenase assay, as specified in the section “Materials and Methods.” [file Image_8.TIFF]
