# Supplementary material for: Pseudomonas aeruginosa Lipoxygenase LoxA Contributes to Lung Infection by Altering the Host Immune Lipid Signaling
Source: Front Microbiol. 2019 Aug 14;10:1826. doi: 10.3389/fmicb.2019.01826 (PMC6702342; doi:10.3389/fmicb.2019.01826)
Supplement: TABLE S1 — Concentration of metabolites (pg/mg of protein) in human lung epithelial NCI-H292 cells infected or not with PAK’ΔloxA, or PAK’overloxA, at 24 h post-infection. [file Table_1.DOCX]

**Table S1. Concentration of metabolites (pg/mg of protein) in extracts of human lung epithelial NCI-H292 cells infected or not with PAK’*∆loxA*, or PAK’*overloxA* 24 h post-infection**^a^**.**

| **PUFA metabolites** | **NI** | | | | | | |  | **PAK'∆loxA** | | | | | | |  | **PAK'overloxA** | | | | | | |  | **Medium** | | |
| --- | --- | --- | --- | --- | --- | --- | --- | --- | --- | --- | --- | --- | --- | --- | --- | --- | --- | --- | --- | --- | --- | --- | --- | --- | --- | --- | --- |
|  | **Cells** | | |  | **Supernatant** | | |  | **Cells** | | |  | **Supernatant** | | |  | **Cells** | | |  | **Supernatant** | | |  |  |  |  |
| *COX* |  |  |  |  |  |  |  |  |  |  |  |  |  |  |  |  |  |  |  |  |  |  |  |  |  |  |  |
| PGE_2_ | 13146.0 | ± | 1677.0 |  | 1083.0 | ± | 237.3 |  | 5820.0 | ± | 2043.0 |  | 357.0 | ± | 68.7 |  | 3627.0 | ± | 721.3 |  | 396.6 | ± | 82.8 |  | ND | | |
| 8-isoPGA_2_ | 10393.0 | ± | 1901.0 |  | 519.4 | ± | 111.8 |  | 3941.0 | ± | 1305.0 |  | 107.3 | ± | 17.6 |  | 4879.0 | ± | 1441.0 |  | 130.4 | ± | 24.3 |  | ND | | |
| PGF_2α_ | 132.7 | ± | 75.1 |  | 223.5 | ± | 57.8 |  | 244.8 | ± | 204.9 |  | 43.3 | ± | 9.3 |  | 247.4 | ± | 121.6 |  | 42.5 | ± | 6.1 |  | 204.1 | ± | 20.8 |
| TXB_2_ | ND | | |  | 172.9 | ± | 4.2 |  | ND | | |  | 174.7 | ± | 7.2 |  | ND | | |  | 175.0 | ± | 10.0 |  | 174.1 | ± | 2.6 |
| PGD_2_ | 318.5 | ± | 70.4 |  | 19.8 | ± | 9.1 |  | 101.3 | ± | 64.3 |  | 20.6 | ± | 9.3 |  | 43.8 | ± | 19.2 |  | 27.7 | ± | 13,5 |  | 5,5 | ± | 5,5 |
| 15d-PGJ_2_ | 13.1 | ± | 11.5 |  | 11.0 | ± | 2.6 |  | ND | | |  | 14.3 | ± | 3.1 |  | 1.6 | ± | 1.6 |  | 9.1 | ± | 2.3 |  | ND | | |
| PGE_3_ | 1580.0 | ± | 240.6 |  | 434.0 | ± | 16.7 |  | 525.4 | ± | 164.3 |  | 461.4 | ± | 11.5 |  | 349.8 | ± | 87.9 |  | 446.8 | ± | 20.7 |  | 392.0 | ± | 21.7 |
| 18-HEPE | 1170.0 | ± | 283.3 |  | 281.2 | ± | 37.8 |  | 880.4 | ± | 107.8 |  | 780.7 | ± | 111.2 |  | 1134.0 | ± | 163.4 |  | 870.4 | ± | 104.4 |  | 256.5 | ± | 89.1 |
| *LOX* |  |  |  |  |  |  |  |  |  |  |  |  |  |  |  |  |  |  |  |  |  |  |  |  |  |  |  |
| 9-HODE | 1683.0 | ± | 182.8 |  | 50.5 | ± | 5.7 |  | 1053.0 | ± | 114.6 |  | 92.3 | ± | 11.5 |  | 1743.0 | ± | 467.5 |  | 94.5 | ± | 7.8 |  | 90.0 | ± | 34.6 |
| **13-HODE ^c^** | **2165.0** | **±** | **273.8** |  | **155.1** | **±** | **33.8** |  | **1680.0** | **±** | **251.4** |  | **206.1** | **±** | **26.0** |  | **6496.0** | **±** | **666.9** |  | **4465.0** | **±** | **166.4** |  | **90.5** | **±** | **50.4** |
| 5-HETE | 281.8 | ± | 51.4 |  | 20.5 | ± | 7.4 |  | 527.0 | ± | 101.3 |  | 84.5 | ± | 12.7 |  | 911.6 | ± | 245.5 |  | 139.4 | ± | 12,1 |  | 1330.0 | ± | 850.1 |
| 8-HETE | 329.0 | ± | 329.0 |  | 21.6 | ± | 4.7 |  | 40.7 | ± | 27.1 |  | 332.2 | ± | 34.8 |  | 167.4 | ± | 49.0 |  | 295.6 | ± | 45,0 |  | 183.4 | ± | 119.7 |
| 12-HETE | 0.3 | ± | 0.3 |  | 261.2 | ± | 27.8 |  | ND | | |  | 239.7 | ± | 21.7 |  | ND | | |  | 261.1 | ± | 24.9 |  | 2563.0 | ± | 202.3 |
| **15-HETE** | **2542.0** | **±** | **266.4** |  | **71.2** | **±** | **11.7** |  | **1415.0** | **±** | **389.3** |  | **176.3** | **±** | **33.1** |  | **6982.0** | **±** | **912.8** |  | **3907.0** | **±** | **206.1** |  | **343.9** | **±** | **172.9** |
| **LXA_4_** | ND | | |  | **638.8** | **±** | **248.9** |  | ND | | |  | **1335.0** | **±** | **505.0** |  | ND | | |  | **1365.0** | **±** | **538.3** |  | **82.7** | **±** | **82.7** |
| **17-HDoHE** | **531.1** | **±** | **380.4** |  | **229.5** | **±** | **106.9** |  | **ND** | | |  | **581.4** | **±** | **44.3** |  | **13437.0** | **±** | **1504.0** |  | **30571.0** | **±** | **2735.0** |  | **521,3** | **±** | **278.5** |
| 14-HDoHE | ND | | |  | 268.9 | ± | 21.2 |  | ND | | |  | 670.2 | ± | 39.3 |  | ND | | |  | 596.0 | ± | 49.7 |  | 2674.0 | ± | 847.9 |
| *CYP* |  |  |  |  |  |  |  |  |  |  |  |  |  |  |  |  |  |  |  |  |  |  |  |  |  |  |  |
| **8-9-EET** | 1388.0 | ± | 670.8 |  | ND | | |  | 1246.0 | ± | 387.7 |  | ND | | |  | 1928.0 | ± | 456.1 |  | ND | | |  | 90.3 | ± | 90.3 |

^a^Data are expressed as mean ± SEM (n = 6).

^b^ND, not detected because concentrations were lower than LOD. For induction-fold representation, LOD was inserted as default value.

**^c^** 15-LOX dependent metabolites are indicated in bold.
