# Supplementary material for: Pseudomonas aeruginosa Lipoxygenase LoxA Contributes to Lung Infection by Altering the Host Immune Lipid Signaling
Source: Front Microbiol. 2019 Aug 14;10:1826. doi: 10.3389/fmicb.2019.01826 (PMC6702342; doi:10.3389/fmicb.2019.01826)
Supplement: TABLE S2 — Concentration of metabolites (pg/mg of protein) detected in BALF from mice infected or not with PAK’ΔloxA, or PAK’overloxA, at 24 h post-infection. [file Table_2.DOCX]

**Table S2. Concentration of metabolites (pg/mg of protein) detected in BALF from mice infected or not with PAK’*∆loxA*, or PAK’*overloxA*, 24h post-infection**^a^**.**

| **PUFA metabolites** | **NI** | | | | | | |  | **PAK'*∆loxA*** | | | | | | |  | **PAK'*overloxA*** | | | | | | |
| --- | --- | --- | --- | --- | --- | --- | --- | --- | --- | --- | --- | --- | --- | --- | --- | --- | --- | --- | --- | --- | --- | --- | --- |
|  | **Cells** | | |  | **Supernatant** | | |  | **Cells** | | |  | **Supernatant** | | |  | **Cells** | | |  | **Supernatant** | | |
| *COX* |  |  |  |  |  |  |  |  |  |  |  |  |  |  |  |  |  |  |  |  |  |  |  |
| PGA_1_ | ND | | |  | ND | | |  | 233.5 | ± | 37.8 |  | ND | | |  | 344.6 | ± | 77.1 |  | ND | | |
| PGE_2_ | 197.3 | ± | 52.8 |  | 153.6 | ± | 111.0 |  | 35336.0 | ± | 3908.0 |  | 170.7 | ± | 106.3 |  | 28713.0 | ± | 4369.0 |  | 91.9 | ± | 37.1 |
| 8-isoPGA_2_ | ND | | |  | 19.5 | ± | 12.5 |  | 6167.0 | ± | 783.5 |  | 8.2 | ± | 6.6 |  | 9186.0 | ± | 1402.0 |  | 3.0 | ± | 2.0 |
| PGF_2α_ | 643.5 | ± | 128.6 |  | 271.8 | ± | 211.5 |  | 18686.0 | ± | 1469.0 |  | 91.8 | ± | 45.9 |  | 15627.0 | ± | 1645.0 |  | 51.2 | ± | 16.0 |
| TXB2 | 813.3 | ± | 141.5 |  | 19.2 | ± | 8.0 |  | 20583.0 | ± | 1694.0 |  | 51.1 | ± | 31.8 |  | 17247.0 | ± | 1435.0 |  | 46.6 | ± | 4.5 |
| 6kPGF_1α_ | 127.9 | ± | 127.9 |  | 281.3 | ± | 180.3 |  | 1513.0 | ± | 227.0 |  | 247.8 | ± | 208.0 |  | 1290.0 | ± | 197.4 |  | 20.2 | ± | 11.6 |
| PGD_2_ | 7.8 | ± | 7.8 |  | 12.8 | ± | 12.4 |  | 3884.0 | ± | 548.4 |  | 14.0 | ± | 11.0 |  | 2384.0 | ± | 265.1 |  | 7.5 | ± | 2.8 |
| 15d-PGJ_2_ | 30.1 | ± | 23.1 |  | ND | | |  | 106.3 | ± | 14.3 |  | ND | | |  | 227.4 | ± | 50.7 |  | ND | | |
| PGE_3_ | ND | | |  | 3.8 | ± | 3.8 |  | 1213.0 | ± | 129.9 |  | ND | | |  | 982.5 | ± | 167.1 |  | ND | | |
| 18-HEPE | 460.3 | ± | 460.3 |  | 27.8 | ± | 17.9 |  | 885.9 | ± | 67.7 |  | 9.5 | ± | 3.6 |  | 1307.0 | ± | 339.0 |  | 23.9 | ± | 5.0 |
| *LOX* |  |  |  |  |  |  |  |  |  |  |  |  |  |  |  |  |  |  |  |  |  |  |  |
| 9-HODE | 2867.0 | ± | 558.6 |  | 69.0 | ± | 25.0 |  | 16536.0 | ± | 1192.0 |  | 125.1 | ± | 12.9 |  | 15555.0 | ± | 2344.0 |  | 103.9 | ± | 12.0 |
| **13-HODE ^c^** | **5368.0** | **±** | **978.2** |  | **298.5** | **±** | **164.1** |  | **17626.0** | **±** | **1262.0** |  | **250.7** | **±** | **23.2** |  | **30993.0** | **±** | **3354.0** |  | **1284.0** | **±** | **234.7** |
| 5-HETE | 2696.0 | ± | 1011.0 |  | 5.5 | ± | 5.5 |  | 21095.0 | ± | 6009.0 |  | 48.7 | ± | 12.6 |  | 19480.0 | ± | 5237.0 |  | 55.4 | ± | 7.9 |
| 5-oxo-ETE | 420.5 | ± | 420.5 |  | ND | | |  | 1164.0 | ± | 165.8 |  | ND | | |  | 1094.0 | ± | 357.1 |  | ND | | |
| LTB4 | 196.8 | ± | 128.7 |  | 0.6 | ± | 0.6 |  | 5959.0 | ± | 2344.0 |  | ND | | |  | 6063.0 | ± | 2237.0 |  | ND | | |
| 8-HETE | 189.9 | ± | 86.6 |  | 6.7 | ± | 6.6 |  | 638.2 | ± | 77.6 |  | 14.9 | ± | 4.3 |  | 626.2 | ± | 87.8 |  | 13.6 | ± | 2.1 |
| 12-HETE | 2414.0 | ± | 1656.0 |  | 485.5 | ± | 268.6 |  | 5769.0 | ± | 1787.0 |  | 206.4 | ± | 68.0 |  | 3779.0 | ± | 792.1 |  | 180.2 | ± | 45.1 |
| **15-HETE** | **863.6** | **±** | **242.6** |  | **25.6** | **±** | **16.3** |  | **4341.0** | **±** | **499.8** |  | **33.2** | **±** | **13.6** |  | **10208.0** | **±** | **1222.0** |  | **439.0** | **±** | **78.5** |
| **LXA_4_** | **ND** | | |  | **ND** | | |  | **ND** | | |  | **1.9** | **±** | **0.9** |  | **ND** | | |  | **16.2** | **±** | **2.4** |
| 14-HDoHE | 2306.0 | ± | 1142.0 |  | 51.2 | ± | 15.1 |  | 4786.0 | ± | 1086.0 |  | 156.6 | ± | 53.5 |  | 4398.0 | ± | 1168.0 |  | 151.1 | ± | 33.2 |
| **17-HDoHE** | **1237.0** | **±** | **539.6** |  | **73.2** | **±** | **58.5** |  | **5594.0** | **±** | **833.5** |  | **85.3** | **±** | **38.5** |  | **9690.0** | **±** | **1408.0** |  | **1131.0** | **±** | **184.2** |
| PDx | ND | | |  | 3.0 | ± | 3.0 |  | 71.3 | ± | 14.5 |  | 0.4 | ± | 0.4 |  | 61.8 | ± | 42.4 |  | 0.5 | ± | 0.5 |
| LTB5 | ND | | |  | ND | | |  | 767.7 | ± | 283.7 |  | ND | | |  | 830.0 | ± | 289.4 |  | ND | | |
|  |  |  |  |  |  |  |  |  |  |  |  |  |  |  |  |  |  |  |  |  |  |  |  |
| *CYP* |  |  |  |  |  |  |  |  |  |  |  |  |  |  |  |  |  |  |  |  |  |  |  |
| 5.6-EET | ND | | |  | ND | | |  | 636.3 | ± | 24.2 |  | ND | | |  | 357.3 | ± | 85.9 |  | ND | | |
| 5-6-DiHETE | ND | | |  | ND | | |  | 640.9 | ± | 197.9 |  | ND | | |  | 469.0 | ± | 134.6 |  | ND | | |
| 8-9-EET | 404.6 | ± | 180.6 |  | ND | | |  | 686.8 | ± | 74.9 |  | ND | | |  | 478.5 | ± | 62.3 |  | ND | | |
| 11-12-EET | 729.9 | ± | 461.3 |  | ND | | |  | 1138.0 | ± | 146.3 |  | ND | | |  | 471.6 | ± | 217.7 |  | ND | | |
| 14-15-EET | 207.4 | ± | 62.2 |  | ND | | |  | 180.1 | ± | 41.2 |  | ND | | |  | 151.7 | ± | 54.2 |  | ND | | |

^a^Data are expressed as mean ± SEM (n = 5-7 mice/group).

^b^ND, not detected because concentrations were lower than LOD. For induction-fold representation, LOD was inserted as default value.

**^c^** 15-LOX dependent metabolites are indicated in bold.
