# Supplementary material for: Pseudomonas aeruginosa Lipoxygenase LoxA Contributes to Lung Infection by Altering the Host Immune Lipid Signaling
Source: Front Microbiol. 2019 Aug 14;10:1826. doi: 10.3389/fmicb.2019.01826 (PMC6702342; doi:10.3389/fmicb.2019.01826)
Supplement: TABLE S3 — Concentration of lipoxygenase by-products (pg/mg of protein) after incubating and activating human blood neutrophils (5.106 cells) with heat-inactivated or active recombinant lipoxygenase (0.5 μg) from P. aeruginosa strain PA42A2. [file Table_3.DOCX]

**Table S3. Concentration of lipoxygenase by-products (pg/mg of protein) after incubating and activating human blood neutrophils (5x10^6^ cells) with heat- inactivated or active recombinant lipoxygenase (0.5µg) from *P. aeruginosa* strain PA42A2.**

|  | **PUFA metabolites** |  | **Neutrophils + Heat inactivated LoxA** | | | | | | | | |  | **Neutrophils + active LoxA** | | | | | | | | |
| --- | --- | --- | --- | --- | --- | --- | --- | --- | --- | --- | --- | --- | --- | --- | --- | --- | --- | --- | --- | --- | --- |
|  |  |  | **Cells** | | | | |  | **Supernatant** | | |  | **Cells** | | | | |  | **Supernatant** | | |
| *LOX* | |  |  |  | | |  |  |  |  |  |  |  |  | | |  |  |  |  |  |
|  | 9-HODE |  | 1845 | ± | | | 95.7 |  | 124.6 | ± | 11.51 |  | 1923 | ± | | | 791.6 |  | 135.1 | ± | 13.24 |
|  | **13-HODE** |  | **2058** | **±** | | | **167.7** |  | **257.5** | **±** | **67.7** |  | **346975** | **±** | | | **8318** |  | **5488** | **±** | **2978** |
|  | 5-HETE |  | 459.9 | ± | | | 330.9 |  | 10.9 | ± | 10.9 |  | 578.3 | ± | | | 456.8 |  | 10.8 | ± | 10.8 |
|  | 8-HETE |  | ND | | | | |  | ND | | |  | ND | | | | |  | ND | | |
|  | 12-HETE |  | 20.81 | | ± | 20.81 | |  | ND | | |  | 43.65 | ± | | | 43.65 |  | ND | | |
|  | **15-HETE** |  | **128.8** | **±** | | | **257.6** |  | **242** | **±** | **148.5** |  | **546.6** | **±** | | | **10.7** |  | **1602** | **±** | **1249** |
|  | **17-HDoHE** |  | **ND** | | | | |  | **ND** | | |  | **714.5** | **±** | | | **714.5** |  |  | **ND** |  |
|  | 14-HDoHE |  | 99.4 | | ± | 99.9 | |  | ND | | |  | 577.1 | | **±** | 577.1 | |  | ND | | |

^a^Data are expressed as mean ± SEM of triplicate samples, from two independent experiments with distinct donors.

^b^ND, not detected because metabolite concentrations were lower than LOD. For induction-fold representation, LOD was inserted as default value.

**^c^** 15-LOX-dependent metabolites are indicated in bold.
