# Supplementary material for: Pseudomonas aeruginosa Lipoxygenase LoxA Contributes to Lung Infection by Altering the Host Immune Lipid Signaling
Source: Front Microbiol. 2019 Aug 14;10:1826. doi: 10.3389/fmicb.2019.01826 (PMC6702342; doi:10.3389/fmicb.2019.01826)
Supplement: TABLE S4 — Concentration of metabolites (pg/mg of protein) in BALF from LPS (3 μg/mouse) treated mice co-challenged or not either with heat-inactivated or active recombinant PA42A2 lipoxygenase (5 μg/mouse), 24 h post-challenge. [file Table_4.DOCX]

**Table S4. Concentration of metabolites (pg/mg of protein) in BALF from LPS (3µg/mouse) treated mice co-challenged or not either with heat- inactivated or active recombinant PA42A2 lipoxygenase (5µg/mouse), 24h post-challenge.**

|  | **PUFA metabolites** | **LPS** | | | | | | |  | **LPS + Heat inactivated LoxA** | | | | | | |  | **LPS + active LoxA** | | | | | | |
| --- | --- | --- | --- | --- | --- | --- | --- | --- | --- | --- | --- | --- | --- | --- | --- | --- | --- | --- | --- | --- | --- | --- | --- | --- |
|  |  | **Cells** | | |  | **Supernatant** | | |  | **Cells** | | |  | **Supernatant** | | |  | **Cells** | | |  | **Supernatant** | | |
| *COX* | |  |  |  |  |  |  |  |  |  |  |  |  |  |  |  |  |  |  |  |  |  |  |  |
|  | PGE_2_ | 1.9 | ± | 0.6 |  | 11.0 | ± | 11.0 |  | 3.4 | ± | 1.9 |  | 25.7 | ± | 7.7 |  | 1.5 | ± | 0.5 |  | 25.4 | ± | 17,0 |
|  | PGF_2α_ | 4.2 | ± | 0.8 |  | 21.7 | ± | 12.5 |  | 3.9 | ± | 0.8 |  | 46.0 | ± | 25.9 |  | 3.4 | ± | 0.9 |  | 98.5 | ± | 27,5 |
|  | TXB2 | 17.9 | ± | 2.2 |  | ND | | |  | 17.8 | ± | 4.2 |  | ND | | |  | 12.6 | ± | 3.8 |  | ND | | |
|  | 6kPGF_1α_ | ND | | |  | 271.6 | ± | 170.4 |  | ND | | |  | 104.3 | ± | 35.4 |  | ND | | |  | 141.9 | ± | 51.4 |
|  | PGD_2_ | ND | | |  | 15.3 | ± | 15.3 |  | ND | | |  | 6.6 | ± | 6.6 |  | ND | | |  | 3.9 | ± | 3.9 |
| *LOX* | |  |  |  |  |  |  |  |  |  |  |  |  |  |  |  |  |  |  |  |  |  |  |  |
|  | 9-HODE | 10.3 | ± | 0.8 |  | 43.4 | ± | 4.3 |  | 8.1 | ± | 1.2 |  | 66.5 | ± | 13.6 |  | 10.0 | ± | 2.7 |  | 80.0 | ± | 8,4 |
|  | **13-HODE** | **19.2** | **±** | **1.2^c^** |  | **165.8** | **±** | **20.3** |  | **16.7** | **±** | **2.9** |  | **285.5** | **±** | **13.6** |  | **50.2** | **±** | **14.5** |  | **6989.0** | **±** | **1713.0** |
|  | 5-HETE | 5.1 | ± | 1.2 |  | 178.2 | ± | 78.2 |  | 6.0 | ± | 1.2 |  | 326.7 | ± | 77.2 |  | 7.5 | ± | 1.7 |  | 606.0 | ± | 150,3 |
|  | 8-HETE | ND | | |  | 40.9 | ± | 16.5 |  | 0.5 | ± | 0.3 |  | 111.1 | ± | 33.1 |  | 1.8 | ± | 1.3 |  | 44.4 | ± | 12.5 |
|  | 12-HETE | ND | | |  | 250.4 | ± | 69.8 |  | ND | | |  | 524.2 | ± | 83.0 |  | 2.2 | ± | 1.6 |  | 483.0 | ± | 80.8 |
|  | **15-HETE** | **5.7** | **±** | **0.3** |  | **288.7** | **±** | **22.1** |  | **5.2** | **±** | **1.2** |  | **911.6** | **±** | **404.1** |  | **12.6** | **±** | **2.9** |  | **2861.0** | **±** | **319,8** |
|  | **17-HDoHE** | **1.7** | **±** | **1.7** |  | **79.7** | **±** | **46.1** |  | **ND** | | |  | **ND** | | |  | **25.9** | **±** | **8.0** |  | **10424.0** | **±** | **1920.0** |
|  | 14-HDoHE | ND | | |  | 257.2 | ± | 62.6 |  | ND | | |  | 294.1 | ± | 40.9 |  | ND | | |  | 505.4 | ± | 84.0 |
| *CYP* | |  |  |  |  |  |  |  |  |  |  |  |  |  |  |  |  |  |  |  |  |  |  |  |
|  | 14-15-EET | ND | | |  | 90.9 | ± | 90.9 |  | 1.4 | ± | 0.8 |  | 57.2 | ± | 28.8 |  | 2.0 | ± | 0.9 |  | 42.4 | ± | 25.6 |

^a^Data are expressed as mean ± SEM (n = 4 mice *per* group).

^b^ND, not detected because concentrations were lower than LOD. For induction-fold representation, LOD was inserted as default value.

**^c^** 15-LOX dependent metabolites are indicated in bold.
