# Supplementary material for: Pseudomonas aeruginosa Lipoxygenase LoxA Contributes to Lung Infection by Altering the Host Immune Lipid Signaling
Source: Front Microbiol. 2019 Aug 14;10:1826. doi: 10.3389/fmicb.2019.01826 (PMC6702342; doi:10.3389/fmicb.2019.01826)
Supplement: TABLE S5 — Strains and plasmids used in this study. [file Table_5.DOCX]

**Table S5. Strains and plasmids used in this study**

| **Strains and plasmids** | **Caracteristics** | **Source or reference** |
| --- | --- | --- |
| Strains |  |  |
| *Escherichia coli* |  |  |
| HB101 | supE44, hsdS20, recA13, ara-14, proA2, lacY1, galK2, rpsL20, xyl-5, mtl-1, leuB6, thi-1, Sm^r^ | Boyer and Roulland-Dussoix, 1969 |
| XL-1 blue | *rec*A1 *end*A1 *gyr*A96 *thi-1 hsdR17 supE44 relA1 lac [F´ proAB lacIqZΔM15* Tn*10 (Tetr)].* | Stratagene |
| *Pseudomonas aeruginosa* |  |  |
| PAK | *P. aeruginosa* strain K, wild-type | R. Ramphal |
| PAO1 | laboratory strain, wild-type | R. Ramphal |
| NH57388A | laboratory strain, wild-type | Bayes *et al*, 2016 |
| PAK’ | In-frame partial deletion of pscF from bp 78–213 and in-frame deletion of fliC gene in strain PAK;  T3SS defective, non-motile | Jyot *et al*, 2011 |
| PAK’*∆loxA* | In-frame deletion of *loxA* gene in PAK’ | This work |
| PAK’*overloxA* | PAK’*∆loxA* strain in which *loxA* gene expression cassette from plasmid pUC18T-miniTn7T-*loxA* was inserted into the chromosomal *att* site | This work |
| PAK’*∆loxAluxCDABE* | PAK’*∆loxA* strain in which *luxCDABE* genes expression cassette from plasmid pUC18T-miniTn7T-*lux* was inserted into the chromosomal *att* site | This work |
| M56 | Cystic fibrosis isolate positive for lipoxygenase activity; motile | This work |
| M56*∆loxA* | In-frame deletion of *loxA* gene in M56 | This work |
| M56*overloxA* | M56*∆loxA* strain in which *loxA* gene expression cassette from plasmid pUC18T-miniTn7T-*loxA* was inserted into the chromosomal *att* site | This work |
| M56*∆fliC* | In-frame deletion of *fliC* gene in M56 | This work |

| Plasmids^a^ |  |  |
| --- | --- | --- |
| pEX-18-Gm^r^ | AF047518 ; *oriT^+^sacB^+^*, gene replacement vector with MCS from pUC18; Gm^r^ | Hoang *et al,* 1998 |
| pEX-18-Gm^r^-*∆loxA* | *EcoR*I-*Hind*III insertion of PCR overlap product containing ~0.8bp regions flanking the *loxA* gene; result in in-frame deletion | This work |
| pUC18T-miniTn7T-*lux* | KC848884; suicide vector for shuttling single copies of genes directly to the chromosome via a mini-Tn7 element; aacC1 gene encoding gentamicin resistance marker on Tn7 element; contains oriT for mobilization; P1 integron promoter driven expression of luxCDABE; Amp^r^ Gm^r^ | Damron *et al*, 2013 |
| pEX-18-Gm^r^ | AF047518 ; *oriT^+^sacB^+^*, gene replacement vector with MCS from pUC18; Gm^r^ | Hoang *et al,* 1998) |
| pEX-18-Gm^r^-*∆loxA* | *EcoR*I-*Hind*III insertion of PCR overlap product containing ~0.8bp regions flanking the *loxA* gene; result in in-frame deletion | This work |
| pUC18T-miniTn7T-*lux* | KC848884; suicide vector for shuttling single copies of genes directly to the chromosome via a mini-Tn7 element; aacC1 gene encoding gentamicin resistance marker on Tn7 element; contains oriT for mobilization; P1 integron promoter driven expression of luxCDABE; Amp^r^ Gm^r^ | Damron *et al*, 2013 |
| pUC18T-miniTn7T-*loxA* | pUC18T-miniTn7T-lux vector with the *luxCDABE* genes replaced by *loxA* gene amplified with primer RBS-Lox and STOP-Lox under the control of the P1 integron promoter | This work |
| mini-CTX*lux* | AF25149 ; suicide vector for shuttling single copies of genes directly to the chromosome via fCTX element; contains oriT for mobilization; SMC upstream luxCDABE operon; Tet^r^ | Becher and Schweizer, 2000 |
| mini-CTX*lux*-P*_loxA_* | *Spe*I-*Pst*I insertion of 1kb region located upstream *loxA* gene in multiple-cloning-site of miniCTX*lux* vector | This work |
| pRK2013 | RK2 derivative, Km^r^; self-transmissible | Ditta *et al*, 1980 |
| pTNS3 | Helper plasmid encoding the Tn7 site-specific transposition pathway; Amp^r^ | Choi and Schweizer, 2006 |

^a^Amp^r^ , Gm^r^ and Tet^r^ are resistance to ampicillin, tetracyclin and gentamycin, respectively.

**Strains and plasmids references**

1. Boyer HW, Roulland-dussoix D. 1969. A complementation analysis of the restriction and modification of DNA in Escherichia coli. J Mol Biol 41:459–472.

2. Jyot J, Balloy V, Jouvion G, Verma A, Touqui L, Huerre M, Chignard M, Ramphal R. 2011. Type II secretion system of Pseudomonas aeruginosa: in vivo evidence of a significant role in death due to lung infection. J Infect Dis 203:1369–1377.

3. Hoang TT, Karkhoff-Schweizer RR, Kutchma AJ, Schweizer HP. 1998. A broad-host-range Flp-FRT recombination system for site-specific excision of chromosomally-located DNA sequences: application for isolation of unmarked Pseudomonas aeruginosa mutants. Gene 212:77–86.

4. Damron FH, McKenney ES, Barbier M, Liechti GW, Schweizer HP, Goldberg JB. 2013. Construction of mobilizable mini-Tn7 vectors for bioluminescent detection of gram-negative bacteria and single-copy promoter lux reporter analysis. Appl Environ Microbiol 79:4149–4153.

5. Becher A, Schweizer HP. 2000. Integration-proficient Pseudomonas aeruginosa vectors for isolation of single-copy chromosomal lacZ and lux gene fusions. BioTechniques 29:948–950, 952.

6. Ditta G, Stanfield S, Corbin D, Helinski DR. 1980. Broad host range DNA cloning system for gram-negative bacteria: construction of a gene bank of Rhizobium meliloti. Proc Natl Acad Sci U S A 77:7347–7351.

7. Choi K-H, Schweizer HP. 2006. mini-Tn7 insertion in bacteria with single attTn7 sites: example Pseudomonas aeruginosa. Nat Protoc 1:153–161.
