# Supplementary material for: Pseudomonas aeruginosa Lipoxygenase LoxA Contributes to Lung Infection by Altering the Host Immune Lipid Signaling
Source: Front Microbiol. 2019 Aug 14;10:1826. doi: 10.3389/fmicb.2019.01826 (PMC6702342; doi:10.3389/fmicb.2019.01826)
Supplement: TABLE S6 — Primers used in this study. [file Table_6.DOCX]

**Table S6. Primers used in this study**

| Application | | Sequence (from 5’ to 3’) ^a,b^ |
| --- | --- | --- |
| *Cloning* | |  |
|  | RBS-Lox (*Pst*I) | GAATTCTGCAGGAGGAGACGTGATATGAAACGC**AGGAG**TGTGCT |
|  | STOP-Lox (*Kpn*I) | ATAATTGGTACCTCAGATATTGGTGCTCGCCGGGAT |
|  | *loxA*_5' (*Eco*RI) | CCAATGAATTCATCTGGCGATTCTGGTACTT |
|  | *loxA*_int1 | CGTCCTCTTTCTCCGGCTCACATATCATCGACTCCATC |
|  | *loxA*_int2 | GATGGAGTCGATGATAGTTGAGCCGGAGAAAGAGGACG |
|  | *loxA*_3' (*Xba*I) | CCAATTCTAGACGACAACGCCGTAGTCATCG |
|  | P*_loxA_*-F (*Spe*I) | ATATAACTAGTCAATGAGCGGTCGAGGCGTC |
|  | P*_loxA_*-R (*PstI*) | AGCTACTGCAGTCAcatATCATCGACTCCATC |
| *Insertion control* | |  |
|  | Tn7L | ATTAGCTTACGACGCTACACCC |
|  | Tn7R | CACAGCATAACTGGACTGATTTC |
|  | *glm*S-F | CTGTGCGACTGCTGGAGCTGA |
|  | *glm*S-R | GCACATCGGCGACGTGCTCTC |
| *qRT-PCR* | |  |
|  | *loxA*-F | AACCTGATTCCGCCGAGTTT |
|  | *loxA*-R | ACATTGGCGAAGTTCTCGGT |
|  | *rpoD*-F | GGGCGAAGAAGGAAATGGTC |
|  | *rpoD*-R | CAGGTGGCGTAGGTGGAGAA |
|  | mMIP-1α-F | CCATATGGAGCTGACACCCC |
|  | mMIP-1α-R | TCAGGAAAATGACACCTGGCT |
|  | mIL-6-F | CTGCAAGAGACTTCCATCCAG |
|  | mIL-6-R | AGTGGTATAGACAGGTCTGTTGG |
|  | mKC-F | ACAGGGGCGCCTATCGCCAA |
|  | mKC-R | CAAGGCAAGCCTCGCGACCAT |
|  | mRplpO-F | ATGGGTACAAGCGCGTCCTG |
|  | mRplpO-R | GCCTTGACCTTTTCAGTAAG |

^a^Restriction sites used for cloning are underlined

^b^RBS sequence is noted in bold type
