# Supplementary material for: Pseudomonas aeruginosa Lipoxygenase LoxA Contributes to Lung Infection by Altering the Host Immune Lipid Signaling
Source: Front Microbiol. 2019 Aug 14;10:1826. doi: 10.3389/fmicb.2019.01826 (PMC6702342; doi:10.3389/fmicb.2019.01826)
Supplement: TABLE S7 — Scoring and ranking of LoxA substrates. [file Table_7.docx]

**Table S7. Scoring and ranking of LoxA substrates.^a^**

| **Substrate** | | **Global Rank** | **-cDocker**  **Interaction energy (kcal/mol)** |
| --- | --- | --- | --- |
| **Docosahexaenoic acid** |  | 1 | **63.15** |
| **Eicosapentaenoic acid** |  | 2 | **61.70** |
| **Arachidonic acid** |  | 3 | **61.24** |
| **Linoleic acid** |  | 4 | **60.16** |
| **Linolenic acid** |  | 5 | **59.97** |

**Concerning the three last ligands the ranking** c**learly depends on the scoring function**
